# Optimisation of pyruvate hyperpolarisation using SABRE by tuning the active magnetisation transfer catalyst†
†Electronic supplementary information (ESI) available. CCDC 1957542–1957543. For ESI and crystallographic data in CIF or other electronic format see DOI: 10.1039/c9cy02498k


**DOI:** 10.1039/c9cy02498k

**Published:** 2020-01-28

**Authors:** Ben. J. Tickner, Olga Semenova, Wissam Iali, Peter J. Rayner, Adrian C. Whitwood, Simon B. Duckett

**Affiliations:** a Centre for Hyperpolarization in Magnetic Resonance (CHyM) , University of York , Heslington , YO10 5NY , UK . Email: simon.ducket@york.ac.uk; b Department of Chemistry , University of York , Heslington , YO10 5DD , UK

## Abstract

Hyperpolarisation techniques such as signal amplification by reversible exchange (SABRE) can deliver NMR signals several orders of magnitude larger than those derived under Boltzmann conditions. SABRE is able to catalytically transfer latent magnetisation from *para*-hydrogen to a substrate in reversible exchange *via* temporary associations with an iridium complex. SABRE has recently been applied to the hyperpolarisation of pyruvate, a substrate often used in many *in vivo* MRI studies. In this work, we seek to optimise the pyruvate-^13^C_2_ signal gains delivered through SABRE by fine tuning the properties of the active polarisation transfer catalyst. We present a detailed study of the effects of varying the carbene and sulfoxide ligands on the formation and behaviour of the active [Ir(H)_2_(η^2^-pyruvate)(sulfoxide)(NHC)] catalyst to produce a rationale for achieving high pyruvate signal gains in a cheap and refreshable manner. This optimisation approach allows us to achieve signal enhancements of 2140 and 2125-fold for the 1-^13^C and 2-^13^C sites respectively of sodium pyruvate-1,2-[^13^C_2_].

## Abstract 2

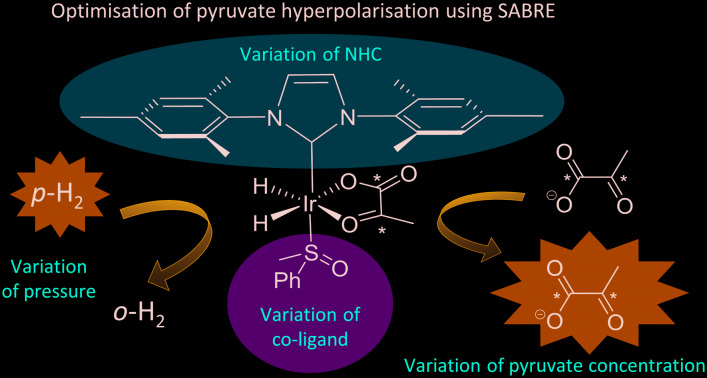
SABRE catalysts [Ir(H)_2_(η^2^-pyruvate)(sulfoxide)(NCH) transfer magnetisation from *para*-hydrogen to pyruvate yielding hyperpolarised ^13^C NMR signals enhanced by >2000-fold. Properties of the catalyst control efficiency.

## Introduction

Nuclear magnetic resonance (NMR) and magnetic resonance imaging (MRI) are some of the most widely used tools for the characterisation of molecules and the clinical diagnosis of disease. While these techniques are used widely in their fields, they remain insensitive as their signal strengths are derived from small Boltzmann population differences across the nuclear spin energy levels they probe. Recently, a growing number of researchers are turning their attention to hyperpolarisation to help address this problem.^1^–^3^ For example, dynamic nuclear polarisation (DNP) can achieve polarisation levels of 92% and 70% for ^1^H and ^13^C signals in times as short as 150 seconds and 20 minutes respectively.^4^,^5^ DNP transfers the inherent polarisation of an electron into target nuclei when both are located in a frozen glass matrix and subject to microwave irradiation at or near the resonance frequency of the electron at very low temperatures (1–2 K).^4^–^6^ Rapid heating of such solids then generates materials that yield MR signal enhancements in solution of up to 5 orders of magnitude.^5^,^6^ This approach has been applied to the production of hyperpolarised biomolecules such as pyruvate,^7^–^14^ succinate,^15^,^16^ and fumarate^17^,^18^ which are then injected and detected *in vivo* alongside their metabolic by-products. Imaging the formation of such metabolites provides a route to studying biochemical tissue function in real time with obvious benefits for disease diagnosis.^7^–^17^

*Para*-Hydrogen induced polarisation (PHIP) methods are potentially a faster and cheaper alternative to DNP.^19^–^21^ The feedstock of PHIP is *para*-hydrogen (*p*-H_2_), which is the isomer of dihydrogen that exists as a nuclear spin singlet. In the first generation of PHIP studies, *p*-H_2_ was typically incorporated into a substrate *via* a hydrogenation reaction.^22^,^23^ The resulting product detection by NMR has since provided many significant observations in the field of catalysis wherein reaction intermediates are detected.^24^–^26^ The catalytic production of hyperpolarised probes suitable for *in vivo* study using this version of PHIP was therefore limited to biomolecules that have facile access to their dehydro-precursor.^15^,^27^,^28^ This limitation has been elegantly alleviated using a variant of PHIP, termed *para*-hydrogen induced polarisation by side arm hydrogenation (PHIP-SAH), which can produce aqueous solutions of hyperpolarised pyruvate and acetate.^29^ In the precursor, pyruvate is functionalised as an ester with an unsaturated side arm which, after hydrogenation by *p*-H_2_ and a magnetic field cycling step to transfer polarisation into the modified pyruvate, can be rapidly released through simultaneous hydrolysis and phase separation.^29^,^30^ The resulting pyruvate can then be detected by MRI through a much stronger, hyperpolarised, response. Hyperpolarised pyruvate prepared in this way is the result of a one-shot, irreversible batch synthesis.

**Fig. 1:**
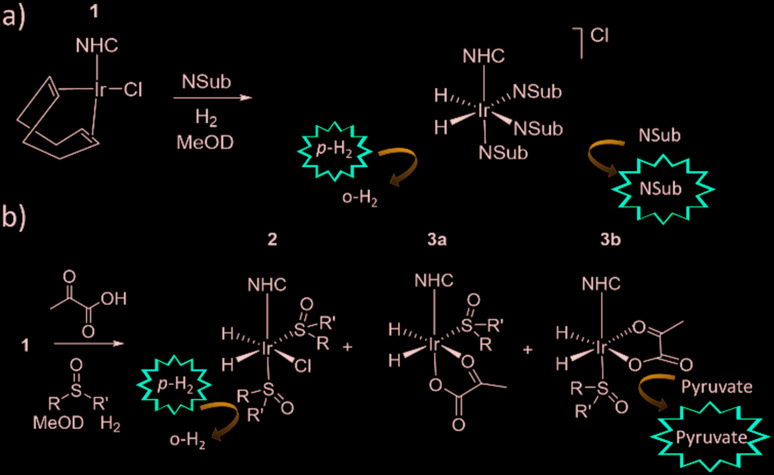
a) Traditionally, SABRE catalytically transfers magnetisation from *p*-H_2_ to an N-donor substrate (NSub) through a temporary *J*-coupled network when both *p*-H_2_ and NSub are in reversible exchange with a complex such as [Ir(H)_2_(IMes)(NSub)Cl b) pyruvate hyperpolarisation using SABRE can be achieved *via* [Ir(H_2_)(η^2^-pyruvate)(DMSO)(IMes)] where *p*-H_2_ exchange is now predominantly mediated by [IrCl(H_2_)(DMSO)_2_(IMes)].

In contrast, signal amplification by reversible exchange (SABRE) is an alternative non-hydrogenative PHIP based method that involves the transfer of spin polarisation from *p*-H_2_ to a substrate when both are concurrently bound to an iridium catalyst, as depicted in Fig. 1.^31^ As the ligands are in reversible exchange, a pool of hyperpolarized substrate is readily created in solution. Hence, the magnetisation transfer step is catalytic in nature, occurring *via* the temporary *J*-coupled network within the organometallic complex. Consequently, the process is completed without chemical change and is continuous and refreshable in nature.^32^ The identity of the ligands used in such SABRE magnetisation transfer catalysts are important in delivering high MR signal gains and controlling the type of substrates that can be hyperpolarised.^33^–^35^ SABRE has had the greatest success to date in polarising structures with N-heterocyclic motifs which have a simple and readily understandable binding mode.^34^,^36^–^38^ In these cases, polarisation transfer catalysts of the form [Ir(H)_2_(NHC)(Sub)_3_]Cl provide suitable substrate (Sub) and H_2_ exchange rates for significant polarisation build up in solution.

Until recently α-keto acids, such as pyruvate, were incompatible with SABRE because they were unable to form stable complexes due to their weak ligation to iridium.^39^ A related technique, SABRE-Relay, has allowed the hyperpolarisation of a wide range of non-ligating substrates that contain functional groups which can receive hyperpolarised protons through exchange from a suitable carrier.^40^,^41^ When applied to sodium pyruvate-1-[^13^C] this approach readily delivers 50-fold ^13^C enhancements.^40^ However, rapid *in situ* condensation of pyruvate with the amine carrier forms products of the type [Ir(H)_2_(η^2^-α-carboxyimine)(amine)(NHC)] which deactivate the system to further pyruvate polarisation.^42^

It has since been reported that by using appropriate stabilising ligands, SABRE can hyperpolarise pyruvate in a low cost, fast, and reversible fashion that does not involve the technologically demanding equipment of DNP, or the multiple steps of PHIP-SAH.^39^ This is possible due to the formation of the polarisation transfer catalyst [Ir(H)_2_(η^2^-pyruvate)(DMSO)(IMes)] (where IMes = 1,3-bis(2,4,6-trimethyl-phenyl)imidazol-2-ylidene) when solutions of [IrCl(COD)(IMes)] (**1a**) (where COD = *cis*,*cis*-1,5-cyclooctadiene), DMSO and sodium pyruvate in methanol-*d*_4_ or 70 : 30 mixtures of D_2_O and ethanol-*d*_6_ are activated with 3 bar of H_2_. These sulfoxide based complexes exhibit significantly more elaborate catalysis than more commonly observed in SABRE with N-donor substrates as the Sub and H_2_ exchange pathways are no longer localised within a single inorganic species.^43^ This is because while [Ir(H)_2_(η^2^-pyruvate)(DMSO)(IMes)] reflects the active polarisation transfer catalyst for ^13^C pyruvate enhancement, it is [IrCl(H)_2_(DMSO)_2_(IMes)] (**2**) that mediates the necessary H_2_ exchange processes.^43^ This situation is complicated yet further by the fact [Ir(H)_2_(η^2^-pyruvate)(DMSO)(IMes)] (**3**) exists as two regioisomers that are differentiated by the geometry of η^2^-pyruvate coordination, as depicted in Fig. 1b. We have previously shown that the regioisomer where pyruvate binds in the same plane as the hydride ligands (**3b**) contains a spin topology that allows active polarisation transfer of singlet order from *p*-H_2_ derived hydride ligands to coordinated ^13^C pyruvate sites in the catalyst.^39^,^43^

SABRE is dependent on the magnetic field experienced by the sample during polarisation transfer because a suitable matching condition for optimal polarisation transfer between *p*-H_2_ derived hydride ligands and the ligated target substrate must be achieved. For ^13^C-SABRE by complexes of this type, optimal transfer typically occurs at mG fields if direct transfer from the *p*-H_2_ derived hydride ligands into bound ^13^C sites is involved.^39^,^43^,^44^ For sodium pyruvate-1-[^13^C] and sodium pyruvate-2-[^13^C] this necessitates fields of ±9 and ±3 mG respectively.^39^ Interestingly, when sodium pyruvate-1,2-[^13^C_2_] is used, the resulting process leads to the spontaneous creation of long lived ^13^C_2_ singlet order in the product being detected whose decoherence lifetime exceeds that of *T*_1_.^39^ In such states, the underlying magnetisation involves two coupled spins and, in this case, its formation is independent of magnetic field. In the context of this paper, it is important to appreciate that *p*-H_2_ reflects another example of such a singlet state which is now not only very long lived, but NMR invisible. This singlet order becomes visible to NMR by a symmetry breaking reaction, such as the oxidative addition of *p*-H_2_ to the iridium centre.^45^ In contrast to *p*-H_2_, the two coupled ^13^C spins of sodium pyruvate-1,2-[^13^C_2_] are already magnetically distinct and consequently its singlet state is immediately accessible by NMR and evolves more quickly that than of *p*-H_2_.

In this paper we report on a series of rigorous catalytic studies that investigate the role that the [Ir(H)_2_(IMes)(η^2^-pyruvate)(sulfoxide)] and [IrCl(H)_2_(DMSO)_2_(IMes)] type species play in the ^13^C hyperpolarisation of pyruvate. Throughout this work we use sodium pyruvate-1,2-[^13^C_2_] as the target substrate because the longer lifetime of its hyperpolarised singlet state may have future benefits in reaction monitoring or medical imaging. It must be remembered that as we create the hyperpolarised molecule remote to the final point of observation there is a time delay between preparation and detection. Thus increased magnetic state lifetimes extend the timescale over which signal detection is possible and offer significant potential benefits for tracer analysis. We show here that optimisation of the hyperpolarisation level delivered by the catalyst is complex, with factors such as catalyst identity, concentration and temperature exhibiting non-trivial behaviour due to the complex interplay that exists between the roles of the different species present in solution. Consequently, we explore the properties of the active catalyst by varying the identity of both sulfoxide and NHC ligand to produce a rationale for achieving high ^13^C pyruvate NMR signal enhancements using SABRE.

## Results and discussion

### Formation of an active sulfoxide containing magnetisation transfer catalyst, [Ir(H)_2_(IMes)(η^2^-pyruvate)(DMSO)]

When sodium pyruvate-1-2-[^13^C_2_] (6 equivalents relative to iridium) is used as the substrate and added to **1** in the presence of dimethyl sulfoxide (DMSO) (4 equivalents) and 3 bar H_2_ in methanol-*d*_4_, an equilibrium mixture of [IrCl(H)_2_(sulfoxide)_2_(NHC)] (**2**) and [Ir(H)_2_(η^2^-pyruvate)(sulfoxide)(NHC)] (**3**) is formed (Fig. 1b).^39^,^43^ The regioisomer containing ligated pyruvate which lies *trans* to both hydride, and the NHC, is labelled **3a** whereas the regioisomer where pyruvate lies in the same plane as the two hydride ligands is labelled **3b**. Both of these structures are illustrated in Fig. 1b.

**Fig. 2:**
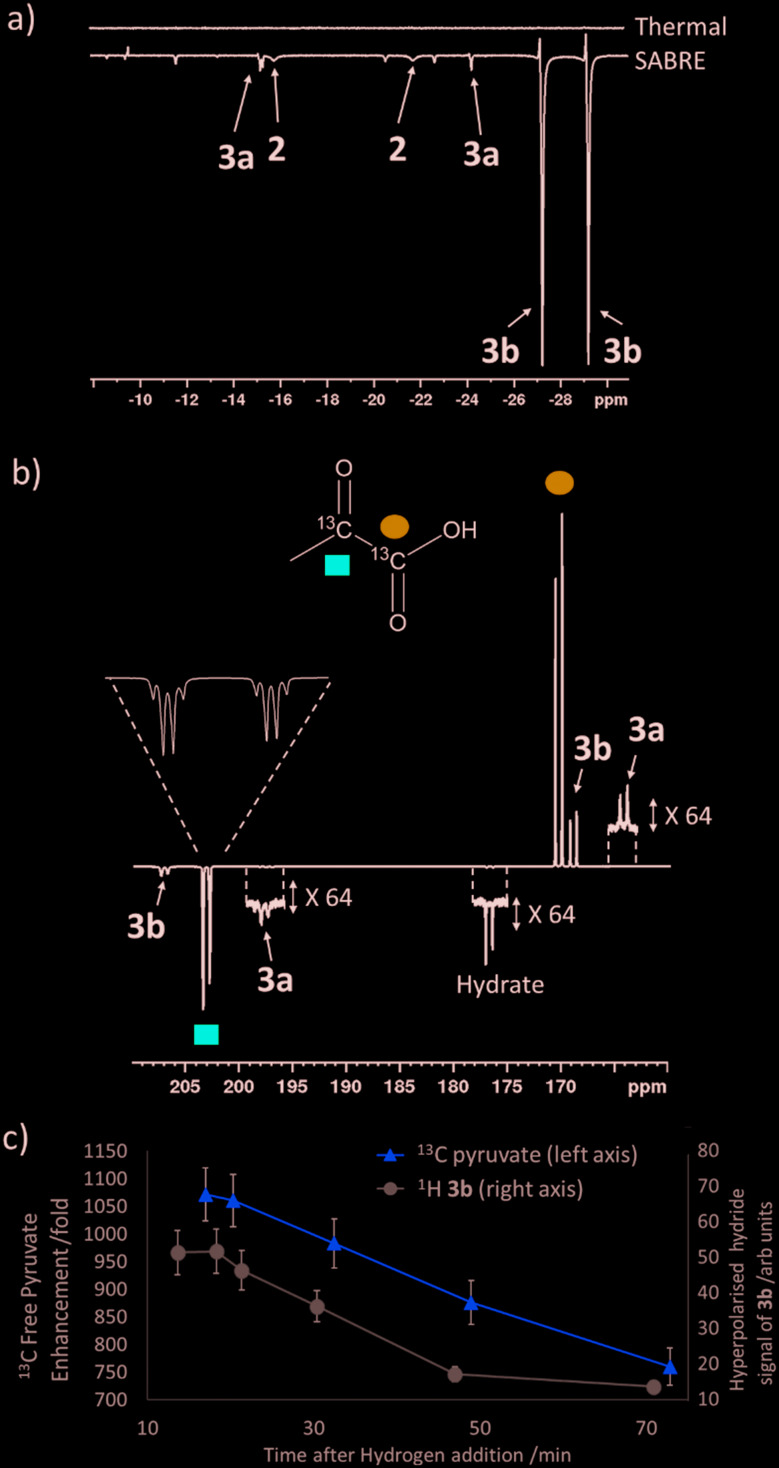
NMR spectra of a) ^1^H hydride region and b) ^13^C carbonyl region after shaking a solution of **1a**, 6 equivalents sodium pyruvate-1,2-[^13^C_2_] and 4 equivalents of DMSO with 3 bar *p*-H_2_ for 10 seconds a) at 65 G or b) in a mu metal shield c) the averaged ^13^C signal gain across the [1-^13^C] and [2-^13^C] sites and hyperpolarised hydride signal intensity of **3b** can be monitored over time with fresh *p*-H_2_ shaking. Note that one anomalous data point was omitted.

When examining these solutions with a signal averaged 32 scan ^1^H NMR measurement at 298 K, the main hydride containing complex present is **3b**. Resonances for **2** and **3a** could not be discerned under these conditions, although, upon shaking this solution with 3 bar of *p*-H_2_ for 10 seconds at 65 G, hyperpolarised hydride responses for **2**, **3a** and **3b** are immediately detected, as shown in Fig. 2a.^39^,^43^ Furthermore, upon shaking this sample for 10 seconds in a mu metal shield (*ca.* 300-fold shielding), hyperpolarised ^13^C resonances are observed, as shown in Fig. 2b. These correspond to those of free pyruvate at *δ* 169 and *δ* 203 (*J*_CC_ = 62 Hz) and pyruvate bound in **3b** at *δ* 168 and *δ* 207 (*J*_CC_ = 60 Hz) and we quantify the ^13^C signal gains as 1215-fold and 910-fold for the [1-^13^C] and [2-^13^C] sites respectively in the free material. Additional resonances corresponding to pyruvate hydrate at *δ* 97 and *δ* 177 (*J*_CC_ = 62 Hz) and pyruvate bound within **3a** at *δ* 166 and *δ* 196 (*J*_CC_ = 63 Hz) are also visible. A multi-scan thermally polarised ^13^C{^1^H} NMR measurement confirms these assignments for ligated pyruvate in both **3a** and **3b** in addition to those of the free material and its hydrate. 2D NMR characterisation data for these complexes has been previously reported.^39^ When we examine the signals of the [1-^13^C] and [2-^13^C] sites more closely we observe ∼2 Hz and ∼20 Hz resonance broadening upon pyruvate coordination respectively. We note that the [2-^13^C] resonance of the free material appears as a doublet of quartets with a ^1^*J*_CC_ value of 62 Hz and a smaller ^2^*J*_HC_ coupling between the adjacent methyl group protons of 6 Hz. This smaller ^2^*J*_HC_ is not visible for pyruvate bound in **3b** due to broadening effects. In order to explore how the efficiency of polarisation transfer changes with reaction time, this sample was shaken with fresh *p*-H_2_ at various time intervals after initial *p*-H_2_ addition. The hyperpolarised ^13^C and ^1^H signals that could be detected for pyruvate and **3b** respectively were found to decrease with time, as shown in Fig. 2c, which is consistent with catalyst decomposition.

### Variation of the [Ir(H)_2_(IMes)(η^2^-pyruvate)(L)] co-ligand, L

Polarisation transfer catalysts of the form [Ir(H)_2_(IMes)(η^2^-pyruvate)(L)] containing η^2^-ligated pyruvate and *p*-H_2_ derived hydride ligands are essential for catalytic polarisation transfer into bound pyruvate ^13^C sites and ultimately free pyruvate after ligand dissociation. A range of different co-ligands, L, (5 equivalents relative to iridium) were screened with [IrCl(COD)(IMes)] (**1a**) (5 mM), and sodium pyruvate-1,2-[^13^C_2_] (6 equivalents), and 3 bar *p*-H_2_ in methanol-*d*_4_ to identify if any other classes of co-ligands besides DMSO could form analogous complexes to **2** and **3**.

The use of 4-chlorobenzenemethanethiol as a co-ligand did not initially yield any hydride containing species. However, upon leaving the solution for a period of several months at 278 K, the growth of single crystals was observed. Upon examination by X-ray diffraction they were found to correspond to [Ir_2_(H)_4_(κ^2^-SCH_2_C_6_H_4_Cl)_2_(IMes)_2_] as detailed in the ESI.† We, and others, have reported structures of similar sulphur bridged iridium dimers^26^,^46^ and other products resulting from SH bond functionalisation.^47^ The other tested co-ligands, formaldehyde, triphenylphosphine (PPh_3_), ethylisothiocyanate and thiophene, all resulted in the formation of hydride complexes within 1 hour of H_2_ addition, but the corresponding solutions did not display any PHIP enhanced hydride signals upon shaking with *p*-H_2_. When a solution of **1a**, PPh_3_ and sodium pyruvate-1,2-[^13^C_2_] with 3 bar H_2_ in methanol-*d*_4_ is left at 278 K for several months, the growth of single crystals was again observed. X-ray diffraction studies identified the product as [Ir(H)_3_(PPh_3_)_3_], as detailed in the ESI.† In contrast, the use of imidazole as a co-ligand did result in a hydride complex at *δ* –22.3 that exhibited PHIP, as detailed in the ESI.† 48,49 However, in each of these cases no additional ^13^C pyruvate resonances were observed by NMR spectroscopy thereby suggesting that pyruvate coordination to iridium in these systems does not occur.

Of the co-ligands tested here, only sulfoxides supported pyruvate ligation to iridium. We expect this to be related to the optimum binding strength of the co-ligand which must be similar to that of pyruvate if its binding is not to be inhibited. For example, when the nitrogen based donor imidazole is used it seems to out compete pyruvate binding.^31^,^34^,^50^–^52^ In these cases [Ir(H)_2_(IMes)(NSub)_3_]Cl type complexes form as revealed by a single hydride signal around *δ* –22.3.^47^,^48^ The use of a sulfoxide based co-ligand is therefore a suitable compromise that leads to pyruvate binding and subsequent ^13^C signal gains and for this reason we explore how its identity affects this process.

### Effect of sulfoxide identity on pyruvate ^13^C_2_**signal** enhancement

**Fig. 3:**
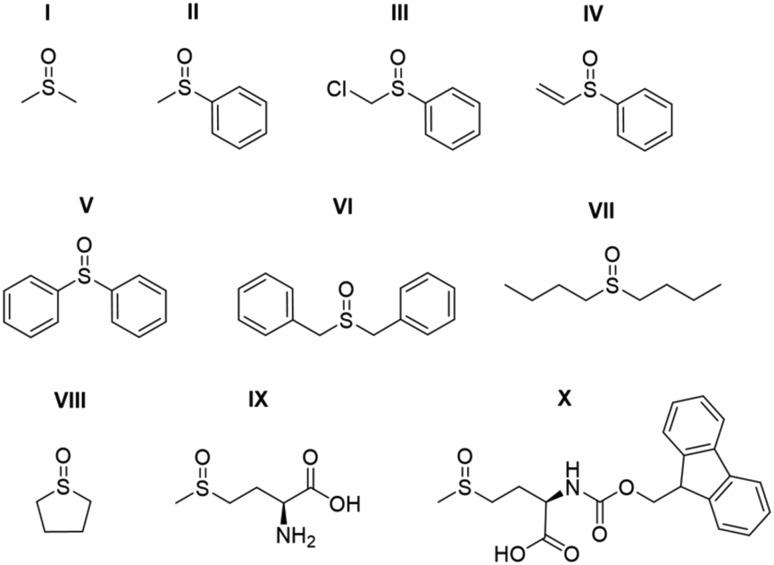
Structures of the sulfoxides **I**–**X** used in this work.

Studies on the effect of sulfoxide identity on the formation of [IrCl(H)_2_(sulfoxide)_2_(NHC)] (**2**) and [Ir(H)_2_(η^2^-pyruvate)(sulfoxide)(NHC)] (**3**) and the subsequent ^13^C pyruvate enhancement proved to be complex. For this work [IrCl(COD)(IMes)] (**1a**) was activated in methanol-*d*_4_ with 3 bar H_2_ in the presence of 6 equivalents of sodium pyruvate-1,2-[^13^C_2_] and 4 equivalents of one of the ten sulfoxides (**I**–**X**) of Fig. 3. The pyruvate ^13^C signal enhancement was then quantified after shaking the sample with fresh *p*-H_2_ several times over a 90 minute time period following H_2_ addition. In order to compare the performance efficiency as a function of sulfoxide identity we define several parameters. The first, *ε*_max_, describes the highest attained free ^13^C pyruvate signal enhancement for either the 1-[^13^C] or 2-[^13^C] site relative to the Boltzmann derived response. We observe that in most cases the signal gain on the 1-[^13^C] and 2-[^13^C] sites are the same within error, and we also quote averaged signal enhancements across the two sites. This is due to creation of ^13^C_2_ singlet order which must be shared equally amongst the two ^13^C sites. The second parameter, *τ*_60_, describes the percentage decrease in ^13^C pyruvate signal enhancement at the 60 minute reaction point when compared to the first measurement. *R***_3b_** is the ratio of the **3b** type product at the *ε*_max_ point relative to the sum of all the hydride containing species and this should illustrate the stability of the sulfoxide–catalyst combination. The relative absolute integrals of the enhanced hydride ^1^H NMR signals of **3b** after shaking at 65 G, *S***_3b_**, was also determined during the reaction period and was found to exhibit similar behaviour. These values are presented for each of the sulfoxides **I**–**X** in Table 1.

**Table 1:** Comparison of *ε*_max_, *τ*_60,_*S***_3b_** and *R***_3b_** values (see text for definition) for methanol-*d*_4_ solutions of **1a**, 6 equivalents of sodium pyruvate-1,2-[^13^C_2_] and 4 equivalents of the specified sulfoxide **I**–**X** of Fig. 3 after shaking with 3 bar *p*-H_2_ for 10 seconds in a mu metal shield.

Sulfoxide	^13^C_1_*ε*_max_/fold^*a*^	^13^C_2_*ε*_max_/fold^*a*^	*τ*_60_/%	*S* **_3b_**/arb. units	*R* **_3b_**/%
**I**	1215 ± 40	910 ± 30	23	50	60
**II**	1090 ± 35	1035 ± 30	6	85	90
**III**	1090 ± 35	1040 ± 30	32	20	93
**IV**	115 ± 5	105 ± 5	92	5	N/A^*b*^
**V**	555 ± 15	545 ± 15	3	60	90
**VI**	195 ± 5	180 ± 5	3	5	70
**VII**	400 ± 10	385 ± 10	9	25	45
**VIII**	1150 ± 35	1040 ± 30	60	70	95
**IX**	0	0	N/A	N/A	0
**X**	130 ± 5	115 ± 5	17	N/A^*c*^	N/A^*c*^

^*a*^These reflect one shot measurements due to the change in signal over time. Errors are based on an average of three measurements for a sample containing **II** where the observed enhancement is relatively constant thereby allowing repeat measurement.

^*b*^Rapid sample degradation prevented recording *R***_3b_** at a similar time point to *ε*_max_.

^*c*^No signals for a species analogous to **3b** were evident.

For sulfoxides **I**–**VIII**, hyperpolarised ^13^C pyruvate responses are observed immediately upon shaking the sample with 3 bar *p*-H_2_ in a mu metal shield. Over the next 90 minute time period, the resulting ^13^C pyruvate signal enhancements all gradually decrease (see ESI†). In all cases, the major dihydride complex present in solution proved to be of type **3b**. Similarly, the hydride signals corresponding to **3b **type products dominate the associated hyperpolarised hydride region of these ^1^H NMR spectra, and the intensity of their signals also decrease with increasing reaction time. Increasing the structural complexity of the co-ligand through the use of amino acid derived sulfoxide, **IX**, resulted in no ^13^C pyruvate signal enhancement, or detection of signals for species of type **3**. While a hydride containing complex forms, which yields resonances at *δ* –12.3 and *δ* –27.3 that exhibit weak PHIP enhancement upon shaking with *p*-H_2_ (see ESI†), no evidence of pyruvate coordination was found. The poor performance of this sulfoxide could relate to the ready formation of an insoluble white precipitate, likely to be the corresponding [pyruvate-COO^–^ + ^+^NH_3_-**IX**] salt. When the protected co-ligand analogue, **X**, is used instead, pyruvate coordination and subsequent enhancement is again observed, but the resulting signal gains are just ∼120 fold. This is consistent with the lack of visible hydride signals for species of type **3**. Hence, we link these low pyruvate enhancements to a low concentration of what we prove later to be the active magnetisation transfer catalyst.

Sulfoxides **I**–**III** and **VIII** delivered the highest levels of ^13^C signal enhancement for pyruvate across this series (*ε*_max_ > 1000 fold) while **IV**–**VII** produced hyperpolarised ^13^C pyruvate signals of lower intensity (100–550 fold). These trends broadly matched those seen for the levels of hydride hyperpolarisation of the corresponding **3b** derivatives. In the case of **IV**, the ^13^C pyruvate response rapidly decayed to zero as a consequence of hydrogenation of the original sulfoxide ligand and subsequent catalyst decomposition which has been observed in closely related systems.^26^ We have reported that C–S bond activation products result from this process which logically accounts for the low *R***_3b_** and *ε*_max_ values achieved by **IV**.^26^ In fact, we suggest that similar sulfoxide reactivity accounts for the loss of the **3b** derivatives in all samples. We highlight that despite a high *ε*_max_ being quantified for **VIII**, catalyst deactivation is extremely rapid. Hence, it is clear that SABRE efficiency is linked to the concentration of the **3b** derivative in solution, which falls as the reaction time increases.

Sulfoxides **VI** and **VII** result in lower proportions of **3b** (*R***_3b_** of 70% and 45% in solution respectively) being present in these mixtures which will contribute to the lower pyruvate signal enhancements that are observed. In contrast, sulfoxides **II**–**V** commonly result in high proportions of **3b** (*R***_3b_** > 90%) and any differences in pyruvate ^13^C signal enhancement between these sulfoxides must now relate more closely to the efficiency of the polarisation transfer catalysis rather than catalyst concentration. For example, we note that **I** achieves a similar level of pyruvate enhancement to **II**, **III** and **VIII** despite the much lower ratio (60%) of **3b** present in solution. In contrast, **IV** and **V** contain similar *R***_3b_** values to **II** and **III** (90%) yet yield much lower pyruvate enhancements (<550-fold as compared to >1000 fold). This suggests that when sulfoxide **I** is utilized, a more effective catalytic system is created when compared to those derived from co-ligands **IV** and **V**.

We conclude from these data that the sulfoxide co-ligand identity plays a significant role in determining the concentration of the active SABRE catalyst in solution and the efficiency of the polarisation transfer process. Thus increasing the proportion of **3b** present in solution is clearly one requirement for optimal SABRE. Of the four sulfoxides that gave pyruvate ^13^C NMR signal enhancements greater than 1000-fold, **II** appeared to be most stable to catalyst decomposition exhibiting only a 6% drop in signal intensity after 1 hour compared to 23, 32 and 60% for **I**, **III** and **VIII** respectively. These results show that H_2_ reaction time is also an important parameter that must be considered when optimising these pyruvate ^13^C NMR signal gains. This is often neglected when polarising N-donor substrates using SABRE as the associated magnetisation transfer catalysts are often more stable over longer reaction times.

Methylphenylsulfoxide, **II**, was identified as the best performing sulfoxide of this series as the associated complex gave some of the highest ^13^C pyruvate signal enhancements whilst also resulting in the highest hyperpolarised hydride ligand signal intensities for the isomer of type **3b**. The concentration of methylphenylsulfoxide **II** was then varied to determine its effect on pyruvate enhancement. Similar behaviour is seen for both the ^1^H and ^13^C NMR signal enhancements of **3b** when compared to those seen for free pyruvate. We find that using 10 equivalents of sulfoxide **II** relative to **1a** provides the highest ^13^C pyruvate response, as shown in the ESI.† We expect that this is related to optimal ligand exchange processes at these effective reagent concentrations.

We have previously reported that complexes of the type [IrCl(H)_2_(NHC)(sulfoxide)_2_] (**2**) exchange hydrogen rapidly and are important in refreshing the *p*-H_2_ derived hydride ligands within the catalytic system.^43^ It is therefore likely that the rate of this process depends on sulfoxide identity and is reflected in the differing hydride signal intensities for the **3b** isomer. The rate of H_2_ exchange within **2** was found to be independent of sulfoxide concentration when the sulfoxide is in excess (6–14 equivalents relative to iridium)^43^ in accordance with the first step of this process being the dissociative loss of sulfoxide. Therefore, we expect that changing sulfoxide concentration must have a greater effect on exchange between **2** and **3**, however this process could not be quantified using EXSY methods. It is clear that the sulfoxide identity plays a role in *p*-H_2_ refreshment within **2** and likely the exchange between **2** and **3**.

### Effect of chloride ions on pyruvate ^13^C_2_**signal** enhancement

**Fig. 4:**
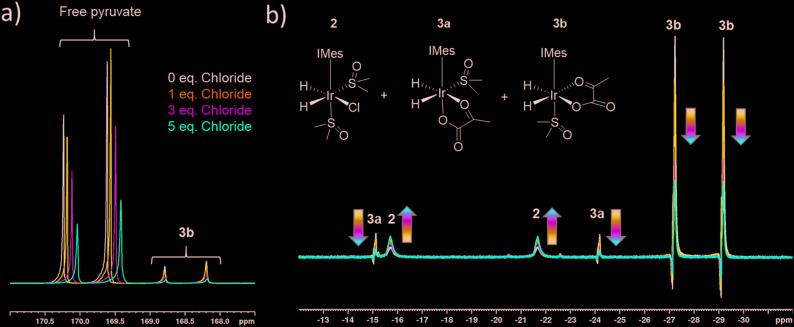
Partial a) ^13^C and b) ^1^H hyperpolarised NMR spectra resulting from shaking a sample of [IrCl(COD)(IMes)], 5 equivalents of sodium pyruvate-1,2-[^13^C_2_], and 10 equivalents of DMSO with varying amounts of NaCl in 0.6 mL methanol-d_4_ with 3 bar *p*-H_2_ for 10 seconds, a) in a mu metal shield 35 minutes following the initial H_2_ addition step and b) at 65 G, 10 minutes following the initial H_2_ addition step.

The rapid rate of H_2_ exchange in **2** in comparison to **3** indicates that **2** provides a clear route to refresh the *p*-H_2_ derived hydride ligands in **3**. It is for this reason that the rate of exchange between **2** and **3** is proposed to play a significant role in determining the observed ^13^C pyruvate signal enhancement. As **2** contains a chloride ligand that is released into solution when **3** is formed, the concentration of available chloride might also be important in the formation of **3**. We have already reported that there is a large decrease in the resulting pyruvate signal enhancement when chloride is replaced by bromide or acetonitrile.^43^ Here, we investigate the effect of changing the chloride concentration. To do this, solutions of **1a** (5 mM), 10 equivalents of sulfoxide **I** and 5 equivalents of sodium pyruvate-1,2-[^13^C_2_] in 0.6 mL methanol-*d*_4_ containing 0–5 equivalents of NaCl in 5 μL of D_2_O were prepared. The resulting SABRE solutions were then activated with 3 bar H_2_ and their ^13^C NMR pyruvate signal enhancements monitored as a function of time. The associated signal intensity *versus* reaction time profiles are given in the ESI† and they all show an initial increase in ^13^C pyruvate signal enhancement over the first ∼30 minute period followed by a subsequent decrease as the reaction time increases. This change mirrors the associated change in concentration of **3b** based on changes in hyperpolarised hydride resonance intensity. Upon increasing the chloride concentration from 0 to 1 equivalents, we observe a decrease in the average pyruvate enhancement across the two sites from 1000-fold to 920-fold. Further decreases to 715-fold and 570-fold are observed as the amount of NaCl is increased to 3 and 5 equivalents respectively. We note that greater chloride concentrations also result in higher proportions of free pyruvate signal relative to that seen for the associated bound resonances within **3b**. These changes are accompanied by an increase in the size of the hydride signals seen for **2** relative to those of **3b**, as shown in Fig. 4. These changes are therefore consistent with a shift in the equilibrium position towards **2** and the resulting fall in pyruvate signal gain is linked to a reduction in the amount of the active polarisation transfer catalyst, **3b** present in these solutions.

The effect of the reduction in active catalyst concentration was tested explicitly by increasing the initial amount of **1a** and **II** so that a greater amount of **3b** was present in solution. The resulting mixture with **1a** (10 mM), **II** (100 mM) and sodium pyruvate-1,2-[^13^C_2_] in a 1 : 10 : 6 ratio yielded averaged ^13^C NMR signal enhancements of 705 and 255-fold for free and bound pyruvate respectively. The corresponding enhancements for the same solution containing 5 equivalents NaCl in 5 μL D_2_O were now much closer for the free pyruvate signal at 690-fold but the bound signal fell to 140 fold. These results confirm that the elevated chloride concentrations increase the proportion of free pyruvate enhancement relative to its bound counterpart. In addition they show that at the 10 mM catalyst concentration the free pyruvate signal enhancement remains comparable to that with the higher NaCl concentration. When the metal concentration was 5 mM, a reduction of pyruvate signal gain upon salt addition takes the averaged signal gain down from 1000-fold to 570-fold. As expected, this difference suggests the greater flux associated with improved efficiency in what would be a bimolecular H_2_ addition step can help offset the effect of increased chloride concentration.

The equilibrium between **2** and **3** is also expected to be influenced by the concentration of pyruvate in solution. Therefore, samples containing **1a**, 10 equivalents of **II** and 3, 6 or 8.5 equivalents of sodium pyruvate-1,2-[^13^C_2_] were shaken with 3 bar *p*-H_2_ in methanol-*d*_4_. Lowering the pyruvate concentration from 6 equivalents to 3 equivalents resulted in the averaged pyruvate signal enhancement reducing from 1085-fold and 515-fold for the free and bound pyruvate respectively to just 770-fold and 365-fold respectively. An increase in pyruvate concentration from 6 to 8.5 equivalents was accompanied by a similar drop in averaged signal gain to 630-fold and 180-fold respectively for the free and bound signals in comparative runs. Interestingly, as the ratio of pyruvate to iridium increases, the proportion of free pyruvate enhancement relative to the bound counterpart in **3b** also increases. This is consistent with an increased likelihood of binding unpolarised pyruvate during SABRE as its concentration increases.

### Effect of catalyst identity on pyruvate ^13^C_2_**signal** enhancement

**Fig. 5:**
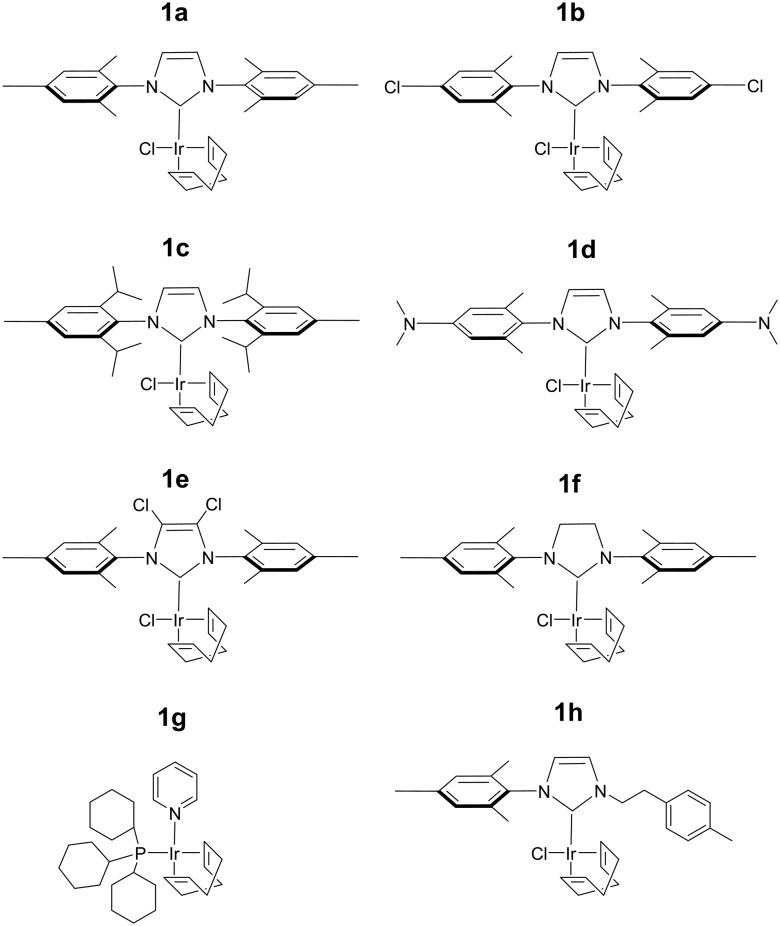
Structures of the eight precatalysts used in this work.

The efficiency of traditional [Ir(H)_2_(NHC)(Sub)_3_]Cl based SABRE catalysts is also influenced by the identity of the NHC ligand in the [IrCl(COD)(NHC)] precatalyst.^34^ Variation of this ligand has been used as a route to optimise signal enhancements by tuning substrate exchange rates.^35^,^53^–^55^ Changes to these ligands has also been used to synthesise water soluble SABRE catalysts.^56^–^58^ The effect of catalyst identity on the pyruvate signal enhancement was therefore probed by investigating the behaviour of the iridium precatalysts, **1a–h**, of Fig. 5. These complexes were chosen to include symmetric N-heterocyclic carbenes with a range of Tolman electronic parameters and % buried volumes.^55^ Additionally, asymmetric N-heterocyclic carbenes^59^ and phosphine containing precatalysts,^31^,^35^ which have both been used previously for SABRE, were also included. Samples were prepared containing 6 equivalents of pyruvate and 10 equivalents of methylphenylsulfoxide (**II**) with 3 bar *p*-H_2_ in 0.6 mL methanol-*d*_4_. The ^13^C pyruvate signal enhancement, ^1^H hydride signal enhancement for the **3b** type product, and its relative proportion in solution as measured in a 32 scan thermal ^1^H NMR spectrum, were monitored periodically over the first 90 minutes of reaction. These values are shown in Table 2 and are displayed graphically in the ESI.†

**Table 2:** Comparison of *ε*_max_, *τ*_60,_ and *R***_3b_** values (see text for definition) measured after shaking a solution of 6 equivalents sodium-1,2-pyruvate-[^13^C_2_] and 10 equivalents **II** with the specified iridium precatalyst **1a–h** of Fig. 5 in methanol-*d*_4_ with 3 bar *p*-H_2_ for 10 seconds in a mu metal shield

Precatalyst	^13^C_1_*ε*_max_/fold^*a*^	^13^C_2_*ε*_max_/fold^*a*^	*R* **_3b_**/%
**1a**	1085 ± 35	1085 ± 35	98
**1b**	915 ± 25	905 ± 25	95
**1c**	905 ± 30	885 ± 25	95
**1d**	980 ± 30	980 ± 30	90
**1e**	650 ± 20	660 ± 20	98
**1f**	870 ± 25	860 ± 25	95
**1g**	35 ± 2	25 ± 2	50
**1h**	60 ± 3	55 ± 3	N/A^*b*^

^*a*^These data reflect one shot measurements due to the change in signal over time. Errors are calculated based on an average of three measurements for a sample containing **1a** where the observed enhancement is relatively constant thereby allowing repeat measurements.

^*b*^No signals for the form corresponding to **3b** were discerned.

The identity of the precatalyst **1a–1f** proved to have little effect on the proportion of the **3b** type product in solution although there was an effect on ^13^C pyruvate signal enhancement. In all cases, a hyperpolarised ^13^C pyruvate response was observed after shaking with 3 bar *p*-H_2_. In contrast to **1a**, precatalysts **1b–1h** result in lower ^13^C pyruvate signal enhancements immediately after H_2_ addition. For some of these precatalysts, *ε*_max_, occurs at much longer reaction times. Hence the differing precatalysts exhibit different activation periods and subsequently, different time points where the maximum concentration of **3b** is reached. This demonstrates how reaction time can be an important parameter that plays a large effect on the observed signal gain. For example, **1a** activates very rapidly and the proportion of its **3b** derivative is at a maximum shortly after the initial H_2_ addition step. The corresponding ^13^C pyruvate signal enhancement is also maximised at this time point. In contrast, **1c** has one of the slowest rates of **3b** derivative formation and hence the corresponding hyperpolarised pyruvate signal increases after the initial H_2_ addition step as a function of the growth in **3b** concentration (see ESI†). Other precatalysts, such as **1b** and **1d**, also form **3b** derivatives which is reflected in an initial increase in pyruvate enhancement followed by a slow decrease as the concentration of **3** falls over a longer timescale. The resulting ^13^C pyruvate signal enhancements can therefore be used as a route to effectively track the concentrations of **3b** type products in solution and hence monitor the conversion of **1** to **3**.

**Fig. 6:**
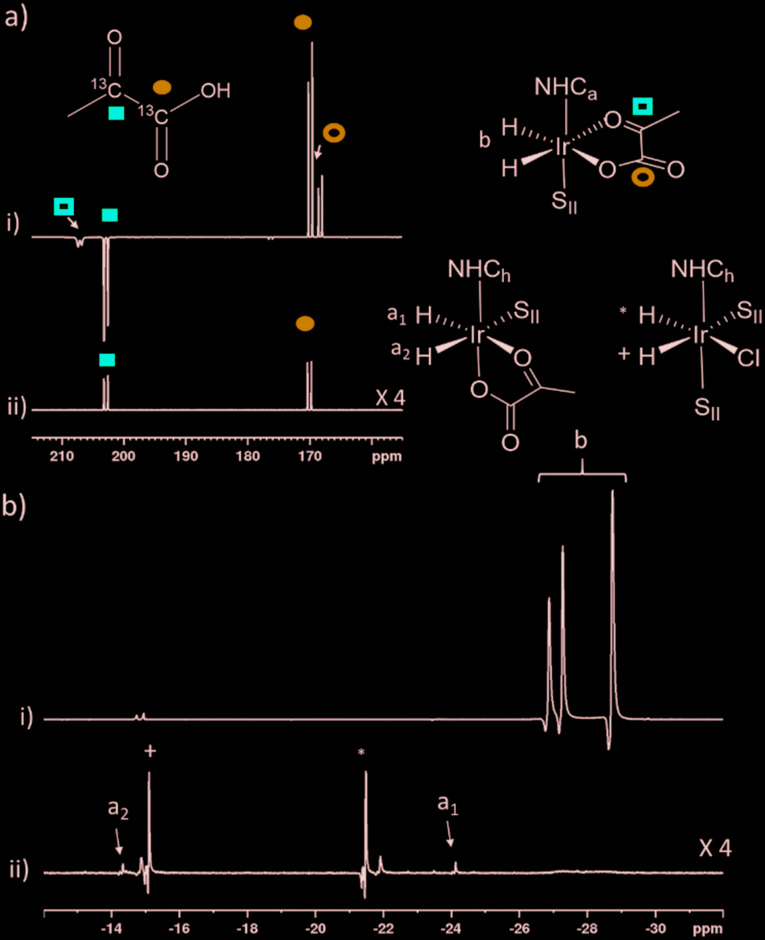
Partial hyperpolarised a) ^13^C and b) ^1^H NMR responses after samples of i) **1a** and ii) **1h** containing 6 equivalents of sodium pyruvate-1,2-[^13^C_2_], and 10 equivalents of **II** are shaken in methanol-*d*_4_ with 3 bar *p*-H_2_ for 10 seconds in a mu metal shield. *S*_**II**_ and *h* in the structures refers to **II** and ligand *h* as shown in Fig. 3 and 5 respectively.

The phosphine based precatalyst **1g** yields just 50% of the **3b** type product in solution and the resulting pyruvate signal enhancements are now just ∼30-fold. In contrast, when precatalyst **1h**, containing an asymmetric N-heterocyclic carbene ligand is used an isomer of type **3b** no longer forms. Here, we form larger amounts of **3a** which we expect to be due to reduced steric crowding associated with the smaller carbene ligand. Now, the hyperpolarised pyruvate signal shows a ∼60-fold enhancement which is an order of magnitude lower than that provided by the symmetric carbene **1a**. This is consistent with the fact isomer **3b** is essential for attaining high levels of pyruvate polarisation. Interestingly, when **3a** is the more dominant species, the hyperpolarised ^13^C_2_ pyruvate profile no longer appears in the typical pattern diagnostic of ^13^C_2_ singlet order as created with catalysts **1a–1g**. This is also consistent with previous theoretical modelling studies which suggest that the spin topology of **3a** is incompatible with the easy retention of *p*-H_2_ derived singlet spin order in the product.^39^ It is clear that **3b**, which is formed when the precatalysts **1a–f** are used, reflects an active polarisation transfer catalysts with the necessary spin topology to mediate efficient polarisation transfer into bound ^13^C_2_ pyruvate. Some examples of representative NMR spectra are shown in Fig. 6.

### Effect of temperature on pyruvate ^13^C_2_**signal** enhancement

**Fig. 7:**
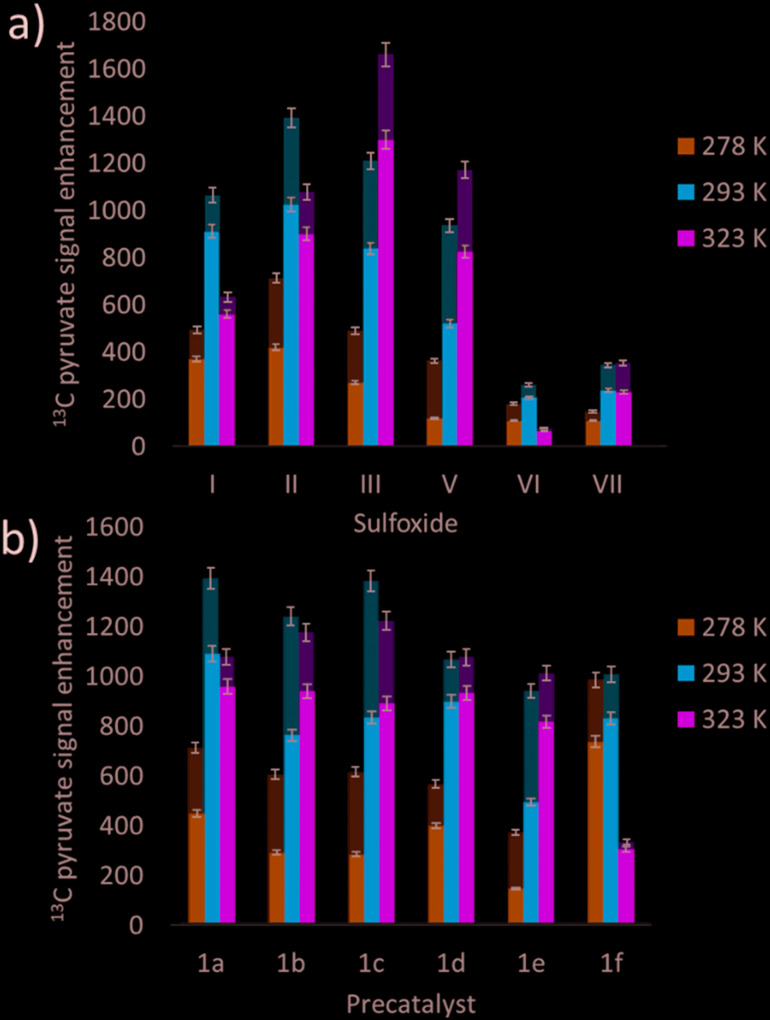
Averaged hyperpolarised free (lower, darker colour) and bound (upper, lighter colour) ^13^C pyruvate NMR signal enhancement values following shaking of methanol-*d*_4_ solutions under SABRE containing a) 4 equivalents of sulfoxide **I**–**III**, **V**–**VII** with **1a** and b) the iridium precatalysts **1a–1f** with 6 equivalents of sodium pyruvate-1,2-[^13^C_2_] and 10 equivalents of II at 3 bar *p*-H_2_ in a mu metal shield after 10 seconds exposure at the specified temperatures.

Many studies have varied the temperature to achieve a substrate exchange rate optimum for SABRE magnetisation transfer.^34^,^41^,^60^ To this end, solutions of **1a**, 6 equivalents of sodium pyruvate-1,2-[^13^C_2_], and 4 equivalents of the sulfoxides **I**–**III**, **IV**–**VII**, shown in Fig. 3, were shaken with 3 bar *p*-H_2_ in methanol-*d*_4_ at three different temperatures. These temperatures (278 K, 293 K and 323 K) were achieved by placing the NMR tube in a thermostatically controlled water bath for 60 seconds prior to shaking for 10 seconds under *p*-H_2_ at room temperature. Care was taken to record the NMR measurements at similar reaction times in order to compare these data. The effect of temperature upon the observed pyruvate ^13^C signal enhancement is shown in Fig. 7a. For **I**, **II**, **VI**, and **VII** the resulting free pyruvate ^13^C signal enhancement is maximised at room temperature while sulfoxides **III** and **V** perform better at the elevated temperature. Hence, whilst ligand exchange is slow and not detectable on the EXSY timescale, there must be an optimum rate for each complex as reported for other N-heterocyclic substrates.^60^

When this study was expanded to include solutions of the iridium precatalysts **1a–f** of Fig. 5, 6 equivalents of sodium pyruvate-1,2-[^13^C_2_], and 10 equivalents of methylphenylsulfoxide (**II**), similar temperature effects were seen as shown in Fig. 7b. For **1a**, 293 K proved optimal with warming clearly moving away from the required exchange rate. For **1b** and **1c**, the proportion of bound substrate polarisation is much higher relative to the free material when compared to the other precatalysts which may suggest slower exchange. This is expected for **1b** on account of the chloride substituent which decreases electron density on the metal relative to **1a** and is therefore likely to result in stronger pyruvate binding and slower ligand exchange. **1c** contains a sterically demanding NHC which might be expected to promote exchange, although this is clearly not the case. In contrast, the metal centre in **1d** is electron rich thereby increasing exchange which agrees with the lower retained bound pyruvate polarisation level. Of the series, **1e** is the most electron deficient with high proportions of bound pyruvate signal and low levels of pyruvate enhancement which increase at higher temperatures. This is consistent with slower pyruvate exchange in the **1e** system.

We conclude that variation of the NHC ligand can have a large effect on the attained pyruvate enhancements. Understanding these effects is challenging and we expect that both steric and electronic effects associated with the ligands are important, as previously suggested for SABRE with N-heterocyclic substrates.^53^,^55^ Here though, the ligand effects are likely to be more complex as they will not only influence the rate of pyruvate exchange within **3**, but also the rate of interconversion between **2** and **3** and the rate of H_2_ exchange in **2**. Steric effects are important in determining the concentration of the active polarisation transfer catalyst, **3b**, that forms in solution. Further optimisation of pyruvate signal gain is possible by subtle variation of the electronic effects of the active catalyst.

### Effect of selective deuteration on pyruvate ^13^C_2_**signal** enhancement

Relaxation within the substrate when bound to the catalyst has been shown to limit its degree of ^1^H hyperpolarisation using SABRE. This effect has been reduced by the inclusion of deuterium labels within the active polarisation transfer catalyst whilst simultaneously reducing any polarisation leakage into the catalyst.^31^,^34^,^61^,^62^ Therefore, we examined the effect of deuterating the sulfoxide ligand and the IMes backbone of the precatalyst.

Shaking a solution of **1a** with 6 equivalents of sodium pyruvate-1,2-[^13^C_2_], 4 equivalents of **I** and 3 bar *p*-H_2_ in methanol-*d*_4_ for 10 seconds in a mu metal shield yielded averaged free and bound pyruvate ^13^C signal enhancements of 1070 and 200-fold respectively. When this process was repeated using the corresponding deuterium labelled sulfoxide **I-*d***_**6**_ at the same time point after H_2_ addition these enhancements remained comparable at 1070 and 220-fold. This suggests that there is little polarisation leakage into this sulfoxide and that relaxation of hyperpolarised magnetisation *via*^1^H sites in its **3b** derivative does not limit the efficiency of SABRE.

In contrast, when the results from shaking a solution of **1a** with 6 equivalents of sodium pyruvate-1,2-[^13^C_2_], and 10 equivalents of **II** under 3 bar *p*-H_2_ in methanol-*d*_4_ are compared to those achieved with **1a**-***d***_**22**_ an effect is seen; in this case all the protons in the NHC except the two in the imidazole ring are labelled with ^2^H. This is reflected in a fall in the averaged free ^13^C_2_ signal enhancement from 1085 to 875-fold although the bound pyruvate signals remain comparable at 515-fold and 535-fold respectively. When this measurement is repeated using **1a**-***d***_**24**_ these signal gains further decrease to 675 and 465-fold for free and bound pyruvate respectively. This suggests that ^2^H labelling of the NHC is now detrimental to SABRE: a finding which is in direct contrast to commonly observed effects when [Ir(H)_2_(NHC)(NSub)_3_]Cl polarisation transfer catalysts are deuterated.^31^,^34^,^61^,^62^ We suggest this could be due to the effects of quadrupolar relaxation at the mG polarisation transfer field caused by the introduction of deuterium, whereas previous studies have typically employed G polarisation transfer fields.

### Further optimisation of pyruvate ^13^C_2_**signal** enhancement by varying shaking time and hydrogen pressure

The effects of the *p*-H_2_ shaking time and hydrogen pressure on the ^13^C pyruvate signal enhancements were also investigated. This involved using a sample of **1a** with two equivalents of **II** at 1, 2 and 3 bar of *p*-H_2_. The results revealed that there was a growth in averaged hyperpolarisation level for free pyruvate from 510 to 730-fold upon increasing the pressure from 1 bar to 3 bar, after which point the signal gain plateaus (see ESI†). This suggests that, in this case, hydrogen exchange is rate limiting at pressures lower than 3 bar but once this pressure is exceeded it is ligand exchange and the associated relaxation processes within the catalyst that become limiting. We have already investigated how hydrogen exchange within [IrCl(H)_2_(IMes)(DMSO)_2_] increases as H_2_ pressure is increased from 0.5–2 bar.^43^ We expect that the increase in pyruvate signal at these H_2_ pressures is related to increased *p*-H_2_ refreshment in the **2**/**3** system which does not become more efficient at pressures higher than 3 bar.

Higher averaged ^13^C_2_ pyruvate signal enhancements result (1050 compared to 425-fold) when the shaking time in the mu metal shield is extended from 5 to 30 seconds in 5 second intervals, as detailed in the ESI.† This change allows polarisation to build up more effectively on both the bound and free ^13^C sites. The observed effect on the ^1^H signals of the hydride resonances in **3b** is the opposite, with the visible signal gains decreasing. This implies more signal is transferred to the ^13^C centres at longer shaking times. We confirmed that these trends are also observed for a sample of **1a**-***d***_**24**_ with ten equivalents of **II**.

### Combining optimisation steps to achieve improved pyruvate ^13^C_2_**signal** enhancement

**Fig. 8:**
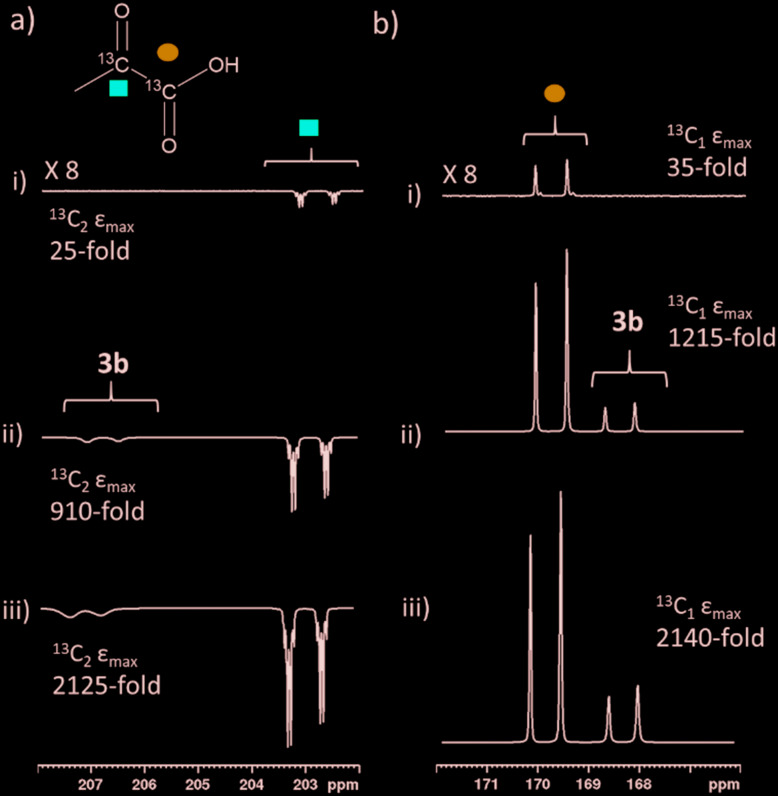
Partial hyperpolarised ^13^C NMR spectra for a) keto region and b) carbonyl region recorded after samples of i) **1g** (5 mM), 10 eq. **II** and 6 equivalents of sodium pyruvate-1,2-[^13^C_2_] ii) **1a** (5 mM), 4 eq. **I** and 6 equivalents of sodium pyruvate-1,2-[^13^C_2_] and iii) **1a** (5 mM), 10 eq. **II** and 6 equivalents of sodium pyruvate-1,2-[^13^C_2_] are shaken in methanol-*d*_4_ with 3 bar *p*-H_2_ for i) and ii) 10 or iii) 30 seconds in a mu metal shield.

We were able to achieve a maximum averaged pyruvate ^13^C signal enhancement of 2135-fold (1.7% polarisation) for the free material alongside a 585-fold bound pyruvate signal gain. This averaged gain corresponds to signal enhancements of 2140 and 2125-fold for the 1-^13^C and 2-^13^C sites respectively. We can therefore increase pyruvate signal gains by two orders of magnitude (from 30-fold for catalyst **1g**, Table 2, to 2135-fold) by careful optimisation of factors including temperature, shaking time, catalyst, sulfoxide and their concentrations. These optimum enhancements involved a sample containing **1a** (5 mM), 10 equivalents **II** and 6 equivalents sodium pyruvate-1,2-[^13^C_2_] in 0.6 mL methanol-*d*_4_ that was shaken with 3 bar *p*-H_2_ for 30 seconds in a mu metal shield. The effect of these optimisation steps on improving the signal gain is depicted in Fig. 8. We failed to see further increases with deuterium labelling of the sulfoxide or the NHC ligands or by shaking with 4.5 bar *p*-H_2_. When all ^13^C species in this sample, including bound pyruvate and its hydrated form, are included a net ^13^C polarisation of 2845-fold (2.3% polarisation) is achieved which exceeds those previously reported.^39^ We also note that the preparations used here are stable for a greater time period after hydrogen addition, thereby allowing for more repeat measurements and improved sample examination.

## Conclusions

Optimising pyruvate signal gains is important for a range of applications that may include hyperpolarised reaction monitoring. For example, we have already demonstrated how the reaction of sodium pyruvate with hydrogen peroxide can be monitored using hyperpolarised ^13^C NMR spectroscopy.^39^ We have presented a detailed study that optimises the pyruvate signal gain by variation of sulfoxide and carbene ligand within [Ir(H)_2_(η^2^-pyruvate)(DMSO)(NHC)] polarisation transfer catalysts. We have shown that sulfoxide co-ligands are essential for the formation of these active species and the highest pyruvate signals can be achieved using a [IrCl(COD)(IMes)] precatalyst in conjunction with 10 equivalents of phenylmethylsulfoxide. Sterically large carbenes are required if the formation of the active isomer **3b** is to be favoured, although electronic effects are important in fine tuning the ligand exchange processes. Hyperpolarised ^13^C_2_ pyruvate signal intensities are shown to be closely linked to the amount of the **3b** isomer present in solution, although systems containing similar amounts but different ligands can result in very different pyruvate enhancements. These results highlight the tension between many different factors that influence the efficiency of polarisation transfer within this complex. In all cases we observe a decrease in both hyperpolarised ^13^C_2_ pyruvate level and the ^1^H hydride ligand signals of the **3b** type isomer at longer reaction times which we associate with catalyst deactivation. By combining these effects we attained an averaged pyruvate ^13^C signal enhancement level of 2135-fold (1.7% polarisation) for free sodium pyruvate-1,2-[^13^C_2_].

For biomedical applications, attaining high signal enhancements in aqueous, rather than methanolic solvents, is of more importance. When a solution of **1a** and 10 eq. **II** are preactivated with 3 bar H_2_ in ethanol-*d*_6_ before adding sodium pyruvate-1,2-[^13^C_2_] in D_2_O and shaking in a mu metal shield for 30 seconds ^13^C signal gains are approximately an order of magnitude lower than those achieved in methanol-*d*_4_. In these 70/30 D_2_O/ethanol-*d*_6_ mixtures, which might reflect a system suitable for future *in vivo* studies, the proportion of bound pyruvate enhancement is significantly higher than those achieved in methanol-*d*_4_. It is therefore clear that in non-methanolic solvents pyruvate exchange is reduced which limits the attained polarisation levels. It is expected that the application of a similar optimisation approach would lead to improved pyruvate enhancements in this biocompatible solvent mixture *via* catalysts that exhibit faster exchange kinetics.

In summary, while SABRE provides a cheap, simple and reversible route to hyperpolarise pyruvate with time and cost advantages over alternative techniques such as DNP and PHIP-SAH, there is a limitation associated with the lower signal enhancements delivered here. Nevertheless, the formation and behaviour of these novel polarisation transfer catalysts and their applications to hyperpolarise pyruvate reflect an important step forward in *para*-hydrogen based hyperpolarisation. Future work is directed at gaining greater understanding of these catalyst effects, probing the ligand exchange processes that govern the attained polarisation levels and optimising them for use in conjunction with biocompatible solvents.

## Experimental

All NMR measurements were carried out on a 400 MHz Bruker Avance III spectrometer using solutions at room temperature (298 K) unless otherwise stated. *Para*-Hydrogen (*p*-H_2_) was produced by passing hydrogen gas over a spin-exchange catalyst (Fe_2_O_3_) and used for all hyperpolarisation experiments. This method produces constant *p*-H_2_ with *ca.* 99% purity. ^1^H (400 MHz) and ^13^C (100.6 MHz) NMR spectra were recorded with an internal deuterium lock. Chemical shifts are quoted as parts per million and referenced to methanol-*d*_4_. ^13^C NMR spectra were recorded with broadband proton decoupling. Coupling constants (*J*) are quoted in Hertz. All commercial compounds listed were purchased from Sigma-Aldrich, Fluorochem, or Alfa-Aesar and used as supplied unless otherwise stated.

Samples were prepared containing 2 mg iridium catalyst with 6 equivalents of sodium pyruvate-1,2-[^13^C_2_] and 4 equivalents of sulfoxide unless otherwise stated in 0.6 mL of methanol-*d*_4_ in a 5 mm NMR tube that was fitted with a J. Young's tap. Unless otherwise stated the iridium precatalyst used was [IrCl(COD)(IMes)] (where IMes = 1,3-bis(2,4,6-trimethyl-phenyl)imidazole-2-ylidene and COD = *cis*,*cis*-1,5-cyclooctadiene). Iridium precatalysts used in this work were synthesized in our laboratory according to literature procedures.^63^ The solutions were subsequently degassed by two freeze–pump–thaw cycles.

The shake & drop method was employed for recording hyperpolarised NMR spectra. NMR tubes were filled with *p*-H_2_ at 3 bar pressure and shaken vigorously for 10 seconds unless otherwise stated in the 65 G stray field next to a 9.4 T magnet for ^1^H polarisation or in a mu metal shield (*ca.* 300-fold shielding) for ^13^C polarisation and placing inside a 9.4 T spectrometer for NMR detection. Pyruvate ^13^C enhancements were calculated by reference to a more concentrated thermal sample as outlined in Shchepin *et al.*^64^ In cases where averaged ^13^C enhancements are given across both 1-[^13^C] and [2-^13^C] sites the sum of these integrals is referenced to the sum of these integrals in the corresponding thermal measurement. Data points usually reflect an average of three shake and drop measurements while those that monitor signal growth over time are single point measurements with typical errors as determined from averaged data.

## Conflicts of interest

B. J. T., W. I., and S. B. D. (and others) are inventors on a patent application filed by the University of York related to this work (patent no. GB1818171.9, filed 7 November 2018).

## Supplementary Material

Supplementary information

Crystal structure data
