# Supplementary material for: Optimisation of pyruvate hyperpolarisation using SABRE by tuning the active magnetisation transfer catalyst
Source: Catal Sci Technol. 2020 Jan 28;10(5):1343–55. doi: 10.1039/c9cy02498k (PMC7315823; doi:10.1039/c9cy02498k)
Supplement: Supplementary file 1 [file CY-010-C9CY02498K-s001.pdf]

Electronic Supplementary Material (ESI) for Catalysis Science & Technology.
This journal is © The Royal Society of Chemistry 2019

Supplementary Material

Optimisation of Pyruvate Hyperpolarisation using SABRE by Tuning the Active Magnetisation Transfer Catalyst

Ben. J. Tickner, Olga Semenova, Wissam Iali, Peter J. Rayner, Adrian C. Whitwood and Simon B. Duckett*

Corresponding Author: simon.duckett@york.ac.uk

Table of Contents

S1. Variation of the [Ir(H)$_2$(IMes)(η^2-pyruvate)(L)] co-ligand, L

S1.1: NMR spectra where L is 4-chlorobenzenemethanethiol
S1.2: NMR spectra where L is Formaldehyde
S1.3: NMR spectra where L is Triphenylphosphine
S1.4: NMR spectra where L is Ethylisothiocyanate
S1.5: NMR spectra where L is Thiophene
S1.6: NMR spectra where L is Imidazole
S1.7: X-ray crystallography of [Ir$_2$(H)$_4$(κ^2-SCH$_2$PhCl)$_2$(IMes)$_2$]
S1.8: X-ray crystallography of [Ir(H)$_3$(PPh$_3$)$_3$]

S2. Monitoring $^{13}C_2$ Pyruvate signal enhancement and 3b concentration over time

S2.1: Effect of changing the sulfoxide
S2.2: Effect of changing the chloride concentration
S2.3: Effect of changing the carbene ligand

S3. Hyperpolarised ^{13}C and ^{1}H NMR spectra

S3.1: Typical hyperpolarised ^{13}C and ^{1}H NMR spectra
S3.2: Hyperpolarised ^{13}C and ^{1}H spectra using sulfoxide IX
S3.3: Hyperpolarised ^{13}C and ^{1}H spectra using sulfoxide X

S4. Optimisation of $^{13}C_2$ Pyruvate signal enhancement

S4.1: Effect of shaking time and *p*-H$_2$ pressure
S4.2: Effect of pyruvate concentration

S5. References

S1. Variation of the [Ir(H)$_2$(IMes)(η^2-pyruvate)(L)] co-ligand, L

Samples were prepared containing [IrCl(COD)(IMes)] (5 mM) (where IMes = 1,3-bis(2,4,6-trimethyl-phenyl)imidazol-2-ylidene and COD = *cis*,*cis*-1,5-cyclooctadiene) with 6 equivalents of sodium pyruvate-1,2-[$^{13}C_2$] and 4 equivalents of the specified co-ligand (L) in 0.6 mL of methanol-d_4 unless otherwise stated in a 5 mm NMR tube that was fitted with a J. Young's tap. The co-ligands used in this study are 4-chlorobenzenemethanethiol, formaldehyde, triphenylphosphine, ethylisothiocyanate, thiophene, imidazole, dimethylsulfoxide (DMSO) (**I**), phenylmethylsulfoxide (**II**), chlorophenylmethylsulfoxide (**III**), vinylsulfoxide (**IV**), diphenylsulfoxide (**V**), dibenzylsulfoxide (**VI**), dibutylsulfoxide (**VII**), tetramethylene sulfoxide (**VIII**), methionine sulfoxide (**IX**) and Fmoc-L-methionine sulfoxide (**X**) which were all purchased from Sigma Aldrich and used without further purification. Unless otherwise stated, the iridium catalyst used was [IrCl(COD)(IMes)]. The iridium precatalysts used in this work were synthesized in our laboratory according to literature procedures.[1] The solutions were subsequently degassed by two freeze-pump-thaw cycles before 3 bar H_2 was added. These samples were then analysed by SABRE-NMR methods. Typical NMR spectra are shown in Figures S1-S24. Some of the data (Figure S9-S20) uses sodium pyruvate-1-[^{13}C] as the reagent due to lower reagent cost.

S1.1: NMR spectra where L is 4-chlorobenzenemethanethiol

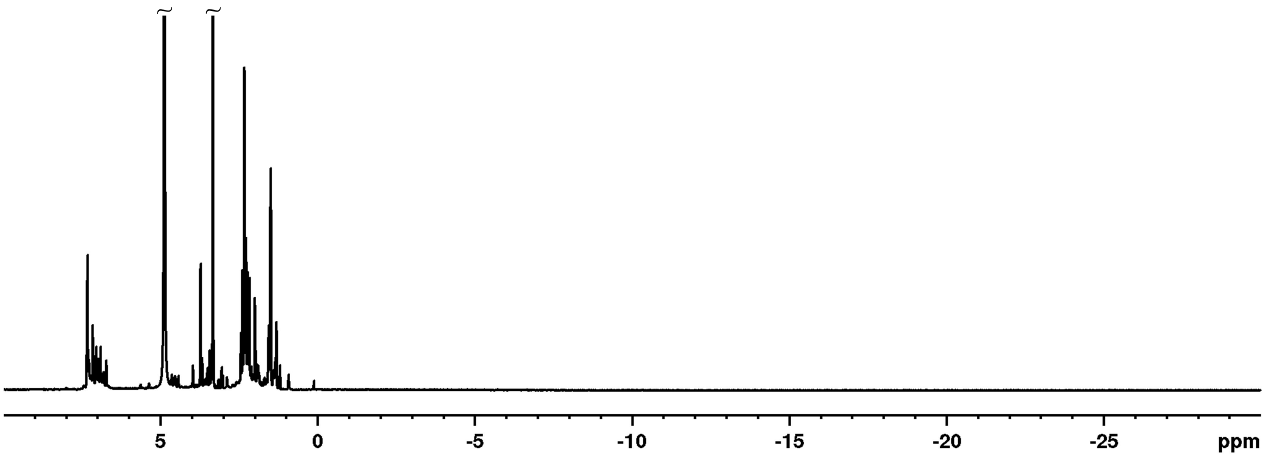

Figure S1: Thermal 64 scan ^{1}H NMR spectrum recorded at 298 K of a sample of [IrCl(COD)(IMes)], sodium pyruvate-1,2-[$^{13}C_2$] and 4-chlorobenzenemethanethiol in methanol-d_4 after leaving for 60 mins in a water bath at 45 °C following the addition of 3 bar H_2

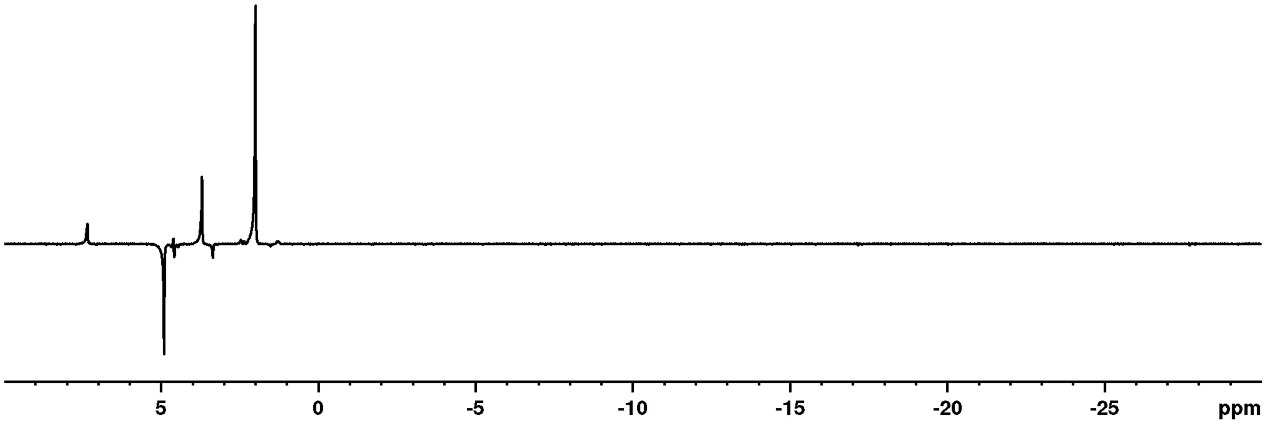

Figure S2: Hyperpolarised 1 scan ^{1}H NMR spectrum recorded at 298 K resulting from shaking a sample of [IrCl(COD)(IMes)], sodium pyruvate-1,2-[$^{13}C_2$] and 4-chlorobenzenemethanethiol in methanol-d_4 with 3 bar p-H_2 for 10 seconds at 65 G.

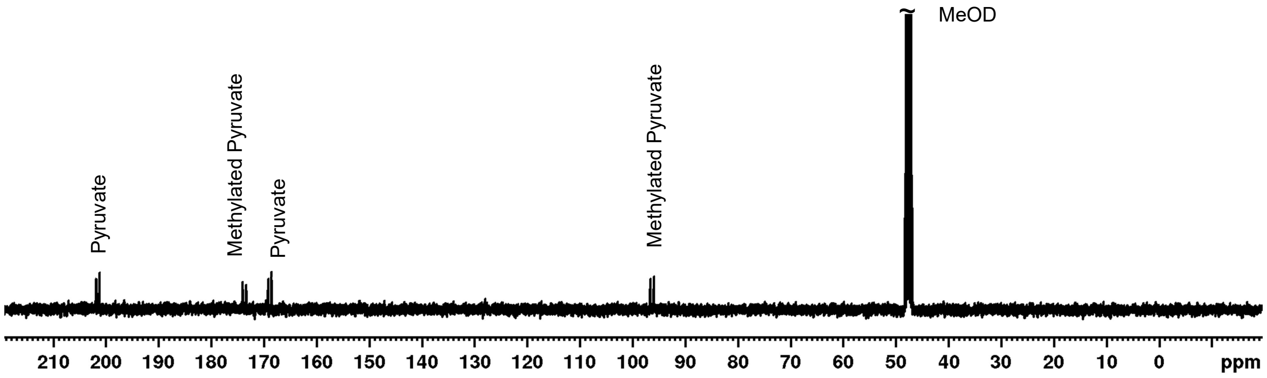

Figure S3: Thermal 128 scan ^{13}C NMR spectrum recorded at 298 K of a sample of [IrCl(COD)(IMes)], sodium pyruvate-1,2-[$^{13}C_2$] and 4-chlorobenzenemethanethiol in methanol-d_4 after leaving the sample for 60 mins in a water bath at 45 °C following the addition of 3 bar H_2

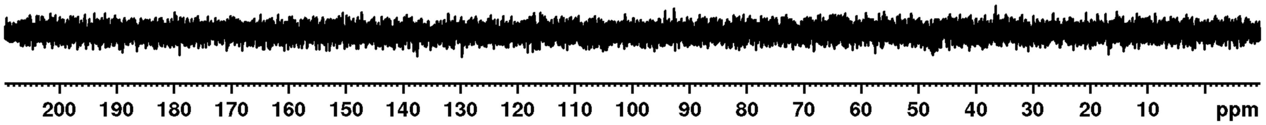

Figure S4: Hyperpolarised 1 scan ^{13}C NMR spectrum at 298 K recorded immediately after shaking a sample of [IrCl(COD)(IMes)], sodium pyruvate-1,2-[$^{13}C_2$] and 4-chlorobenzenemethanethiol in methanol-d_4 with 3 bar p-H_2 for 10 seconds in a mu metal shield.

S1.2: NMR spectra where L is Formaldehyde

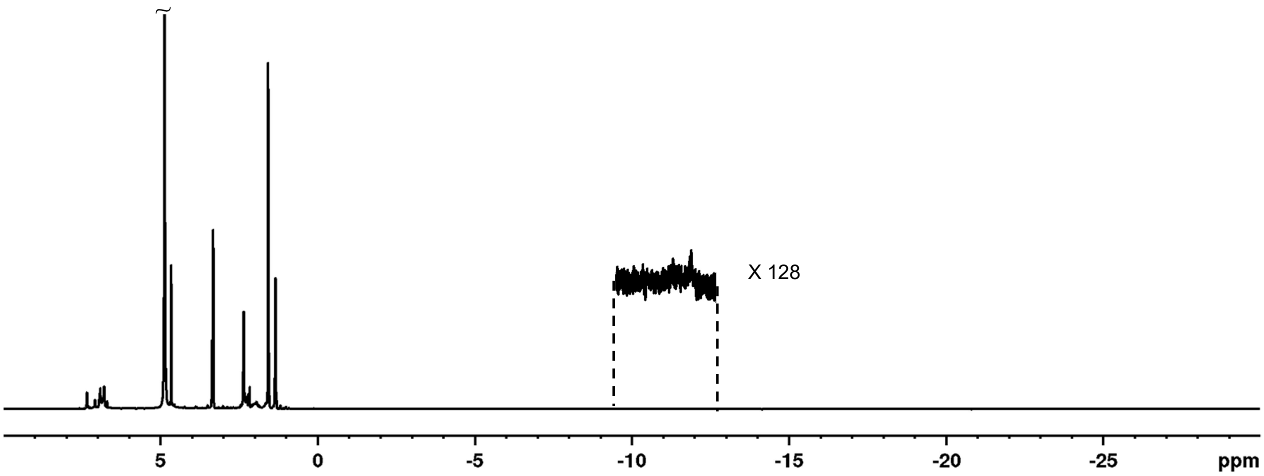

Figure S5: Thermal 32 scan ^{1}H NMR spectrum recorded at 298 K of a sample of [IrCl(COD)(IMes)], sodium pyruvate-1,2-[$^{13}C_2$] and formaldehyde in methanol-d_4 after leaving the sample for 60 mins in a water bath at 45 °C following the addition of 3 bar H_2.

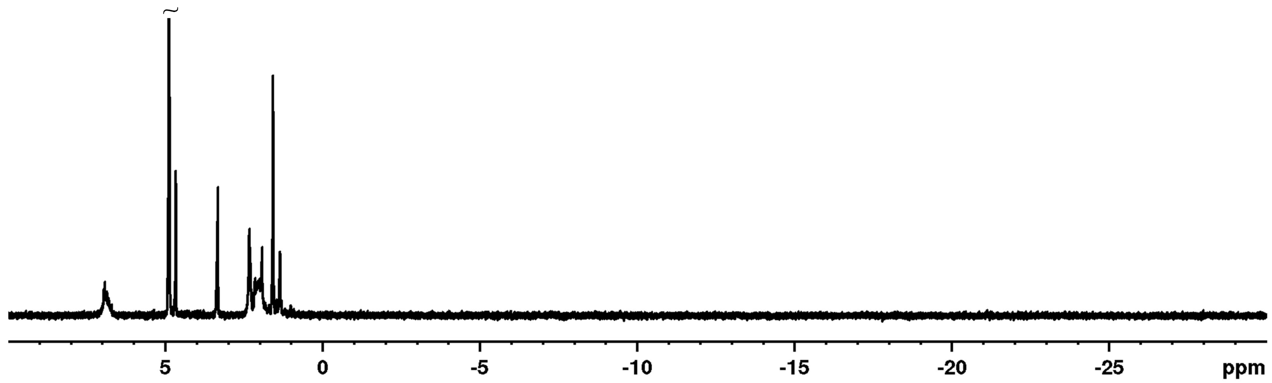

Figure S6: Hyperpolarised 1 scan ^{1}H NMR spectrum recorded at 298 K resulting from shaking a sample of [IrCl(COD)(IMes)], sodium pyruvate-1,2-[$^{13}C_2$] and formaldehyde in methanol-d_4 with 3 bar p-H_2 for 10 seconds at 65 G.

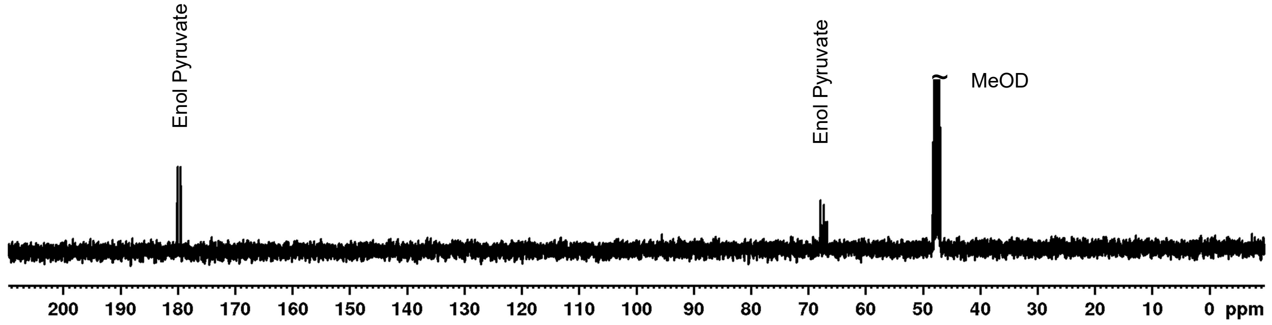

Figure S7: Thermal 64 scan ^{13}C NMR spectrum recorded at 298 K of a sample of [IrCl(COD)(IMes)], sodium pyruvate-1,2-[$^{13}C_2$] and formaldehyde in methanol-d_4 after leaving the sample for 60 mins in a water bath at 45 °C following the addition of 3 bar H_2.

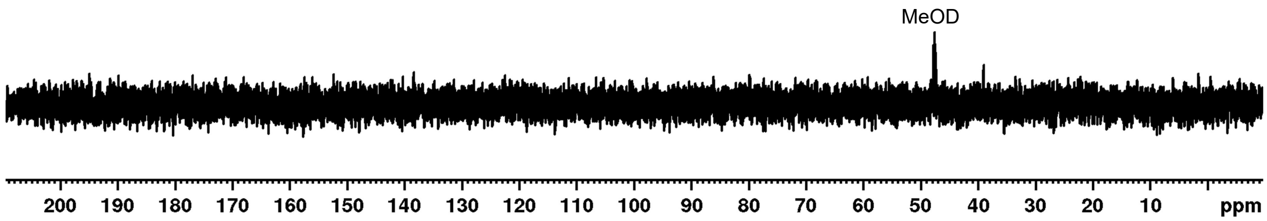

Figure S8: Hyperpolarised 1 scan ^{13}C NMR spectrum at 298 K recorded immediately after shaking a sample of [IrCl(COD)(IMes)], sodium pyruvate-1,2-[$^{13}C_2$] and formaldehyde in methanol-d_4 with 3 bar p-H_2 for 10 seconds in a mu metal shield.

S1.3: NMR spectra where L is Triphenylphosphine

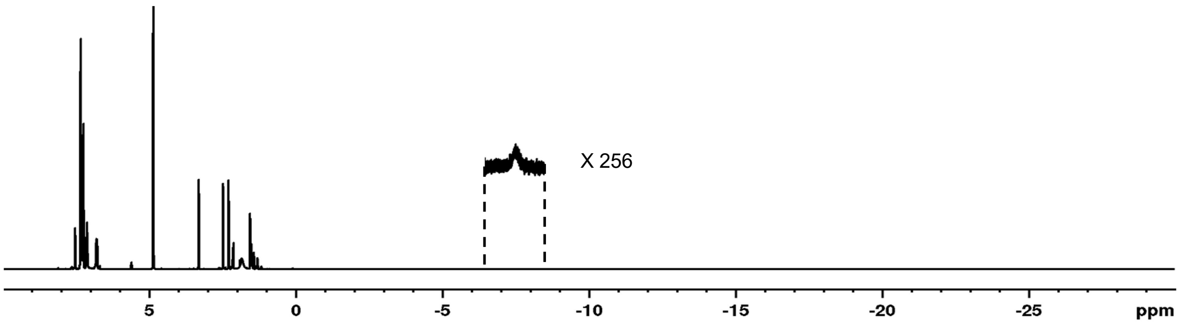

Figure S9: Thermal 32 scan ^{1}H NMR spectrum recorded at 298 K of a sample of [IrCl(COD)(IMes)], sodium pyruvate-1-[$^{13}C_1$] and triphenylphosphine in methanol-d_4 recorded after leaving the sample for 60 mins in a water bath at 45 °C following the addition of 3 bar H_2.

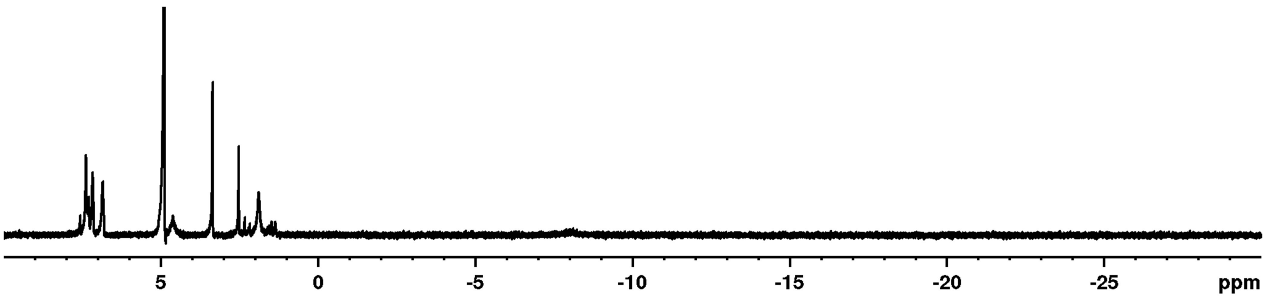

Figure S10: Hyperpolarised 1 scan ^{1}H NMR spectrum recorded at 298 K resulting from shaking a sample of [IrCl(COD)(IMes)], sodium pyruvate-1-[$^{13}C_1$] and triphenylphosphine in methanol-d_4 with 3 bar p-H_2 for 10 seconds at 65 G.

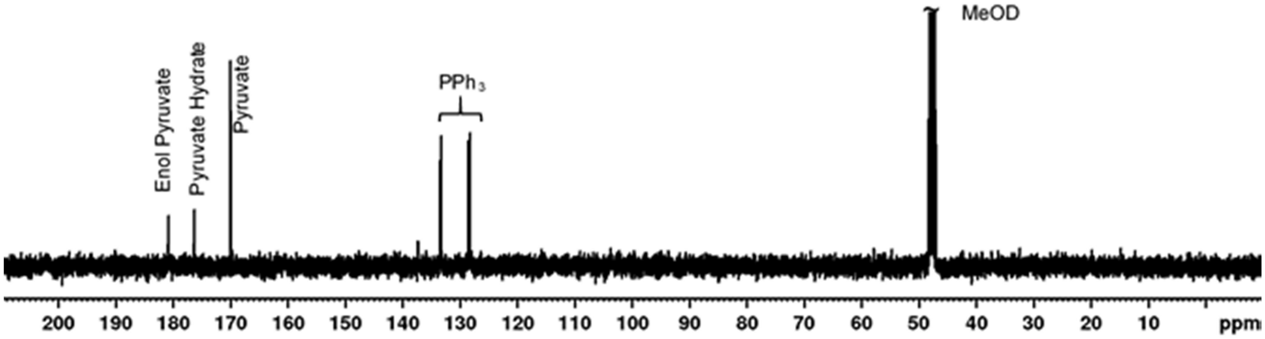

Figure S11: Thermal 64 scan ^{13}C NMR spectrum recorded at 298 K of a sample of [IrCl(COD)(IMes)], sodium pyruvate-1-[$^{13}C_1$] and triphenylphosphine in methanol-d_4 after leaving the sample for 60 mins in a water bath at 45 °C following the addition of 3 bar H_2.

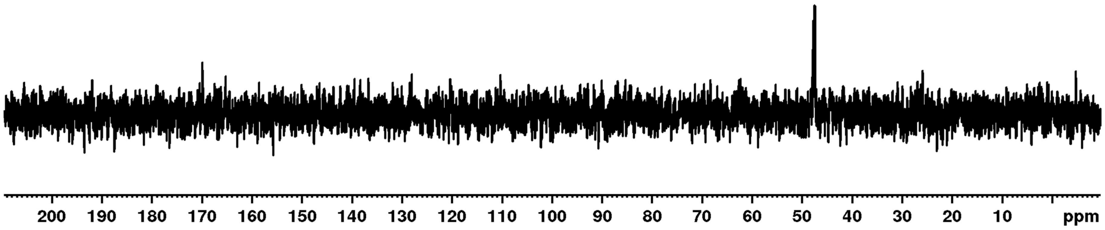

Figure S12: Hyperpolarised 1 scan ^{13}C NMR spectrum recorded at 298 K resulting from shaking a sample of [IrCl(COD)(IMes)], sodium pyruvate-1-[$^{13}C_1$] and triphenylphosphine in methanol-d_4 with 3 bar p-H_2 for 10 seconds in a mu metal shield.

S1.4: NMR spectra where L is Ethylisothiocyanate

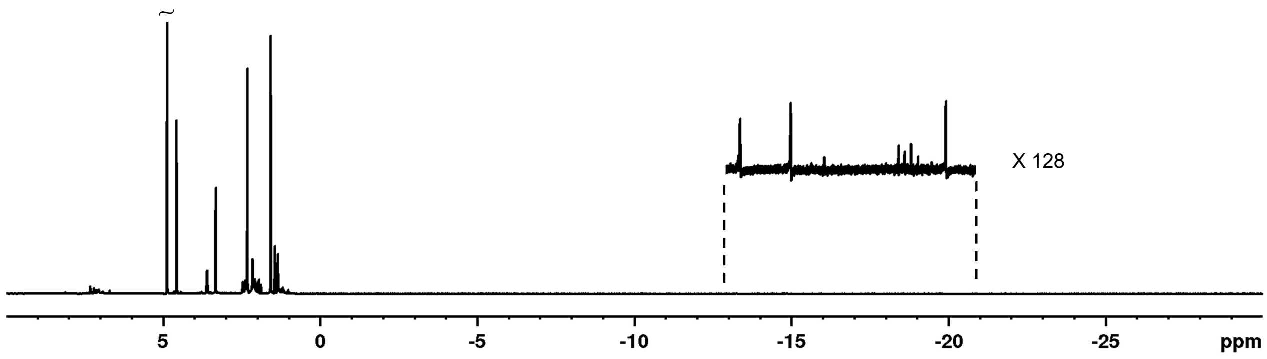

Figure S13: Thermal 64 scan ^{1}H NMR spectrum recorded at 298 K of a sample of [IrCl(COD)(IMes)], sodium pyruvate-1-[$^{13}C_1$] and ethylisothiocyanate in methanol-d_4 after leaving the sample for 60 mins in a water bath at 45 °C following the addition of 3 bar H_2.

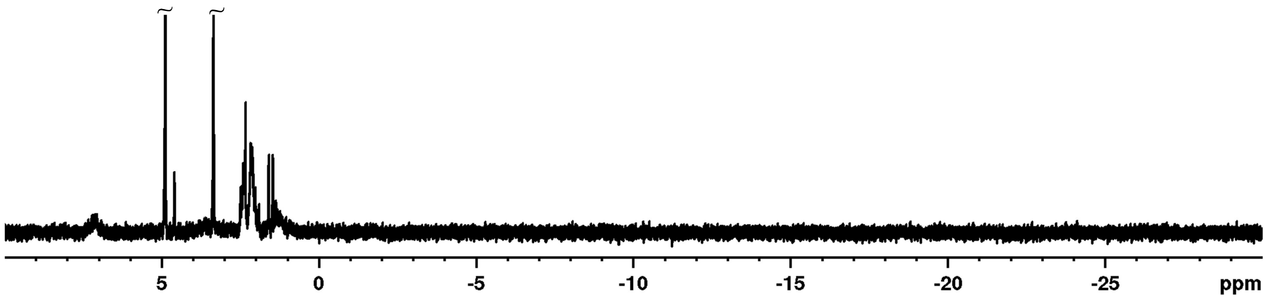

Figure S14: Hyperpolarised 1 scan ^{1}H NMR spectrum recorded at 298 K resulting from shaking a sample of [IrCl(COD)(IMes)], sodium pyruvate-1-[$^{13}C_1$] and ethylisothiocyanate in methanol-d_4 with 3 bar p-H_2 for 10 seconds at 65 G.

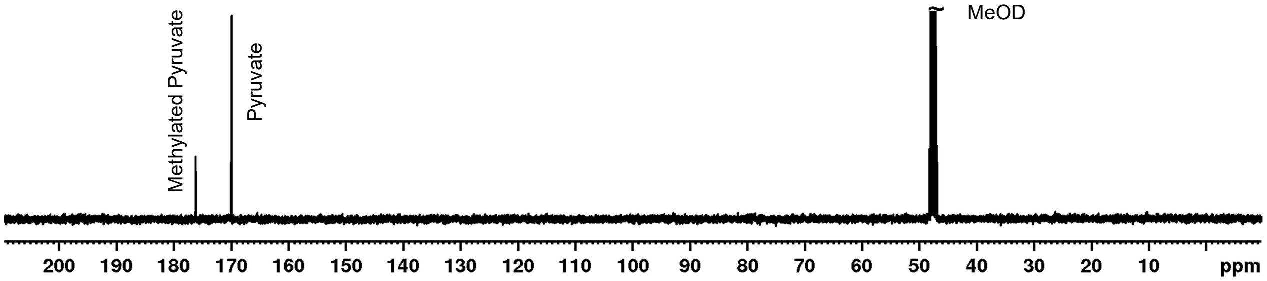

Figure S15: Thermal 64 scan ^{13}C NMR spectrum recorded at 298 K of a sample of [IrCl(COD)(IMes)], sodium pyruvate-1-[$^{13}C_1$] and ethylisothiocyanate in methanol-d_4 after leaving the sample for 60 mins in a water bath at 45 °C following the addition of 3 bar H_2.

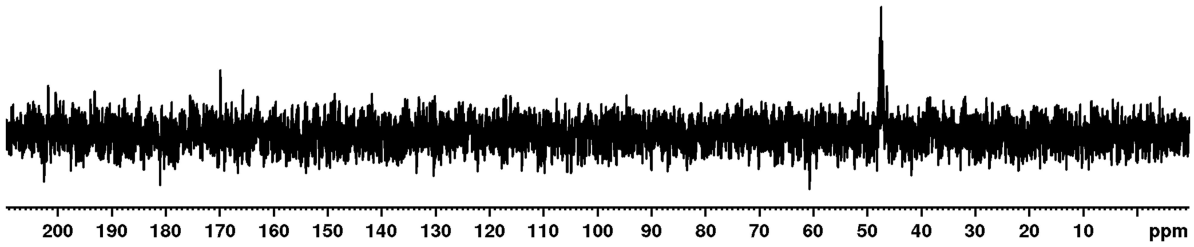

Figure S16: Hyperpolarised 1 scan ^{13}C NMR spectrum recorded at 298 K resulting from shaking a sample of [IrCl(COD)(IMes)], sodium pyruvate-1-[$^{13}C_1$] and ethylisothiocyanate in methanol-d_4 with 3 bar p-H_2 for 10 seconds in a mu metal shield.

S1.5: NMR spectra where L is thiophene

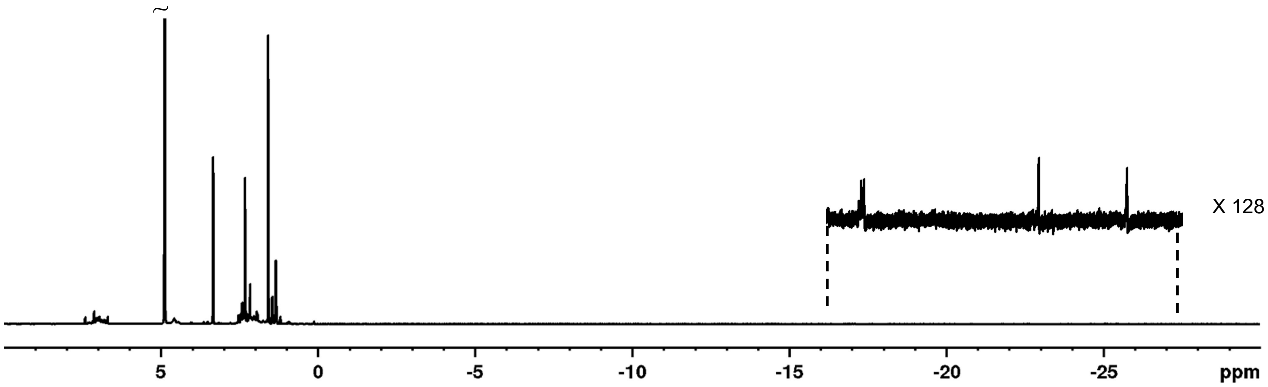

Figure S17: Thermal 64 scan ^{1}H NMR spectrum recorded at 298 K of a sample of [IrCl(COD)(IMes)], sodium pyruvate-1-[$^{13}C_1$] and thiophene in methanol-d_4 after leaving the sample for 60 mins in a water bath at 45 °C following the addition of 3 bar H_2.

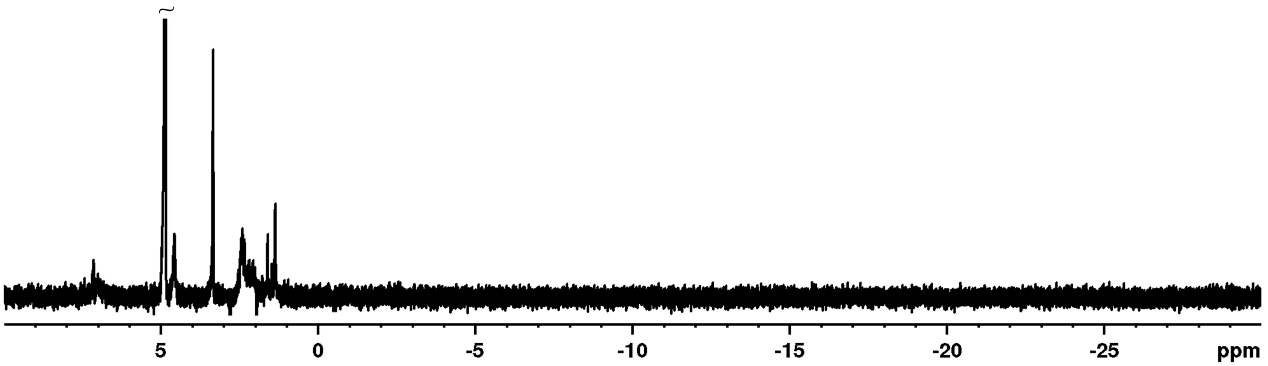

Figure S18: Hyperpolarised 1 scan ^{1}H NMR spectrum recorded at 298 K resulting from shaking a sample of [IrCl(COD)(IMes)], sodium pyruvate-1-[$^{13}C_1$] and thiophene in methanol-d_4 with 3 bar p-H_2 for 10 seconds at 65 G.

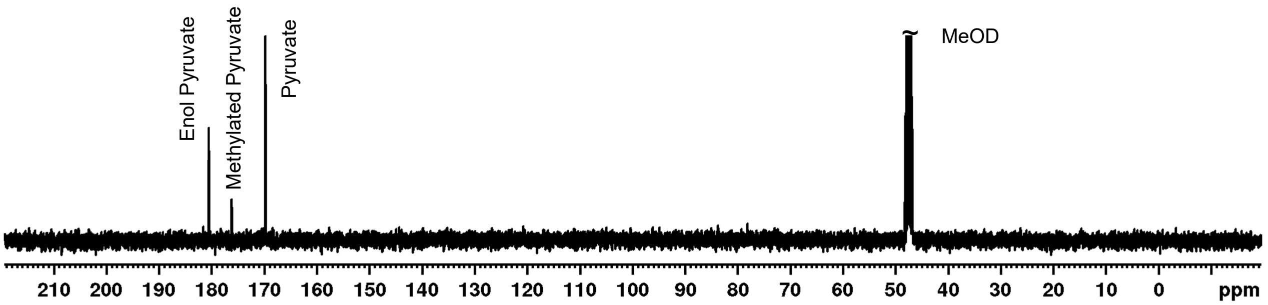

Figure S19: Thermal 64 scan ^{13}C NMR spectrum recorded at 298 K of a sample of [IrCl(COD)(IMes)], sodium pyruvate-1-[$^{13}C_1$] and thiophene in methanol-d_4 after leaving the sample for 60 mins in a water bath at 45 °C following the addition of 3 bar H_2.

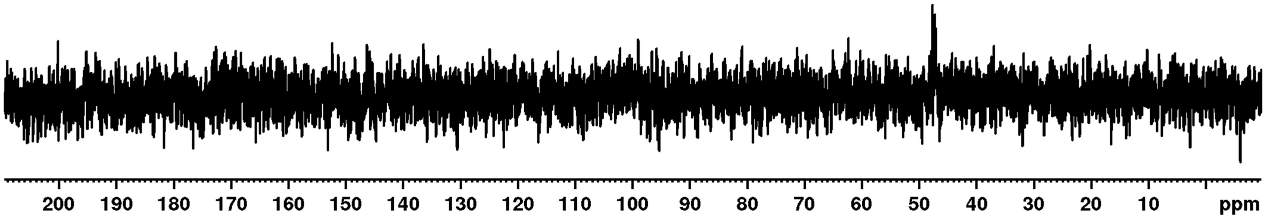

Figure S20: Hyperpolarised 1 scan ^{13}C NMR spectrum recorded at 298 K resulting from shaking a sample of [IrCl(COD)(IMes)], sodium pyruvate-1-[$^{13}C_1$] and thiophene in methanol-d_4 with 3 bar p-H_2 for 10 seconds in a mu metal shield.

S1.6: NMR spectra where L is imidazole

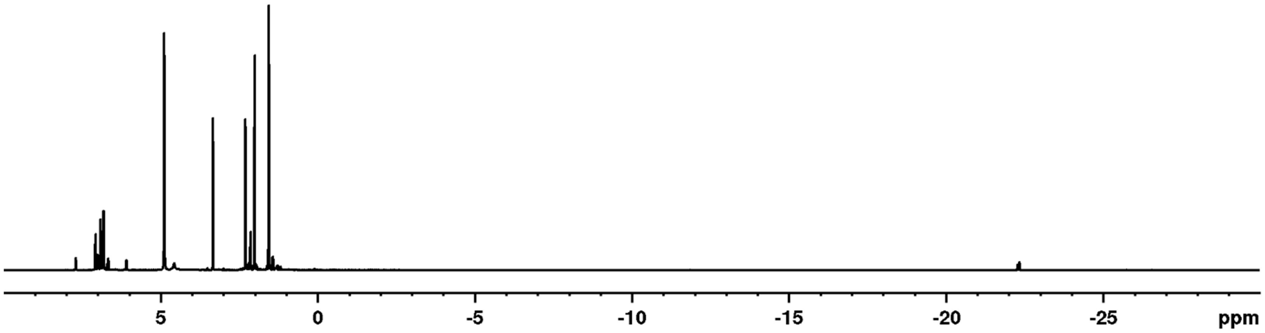

Figure S21: Thermal 64 scan ^{1}H NMR spectrum recorded at 298 K of a sample of [IrCl(COD)(IMes)], sodium pyruvate-1,2-[$^{13}C_2$] and imidazole in methanol-d_4 after leaving the sample for 120 mins in a water bath at 45 °C following the addition of 3 bar H_2.

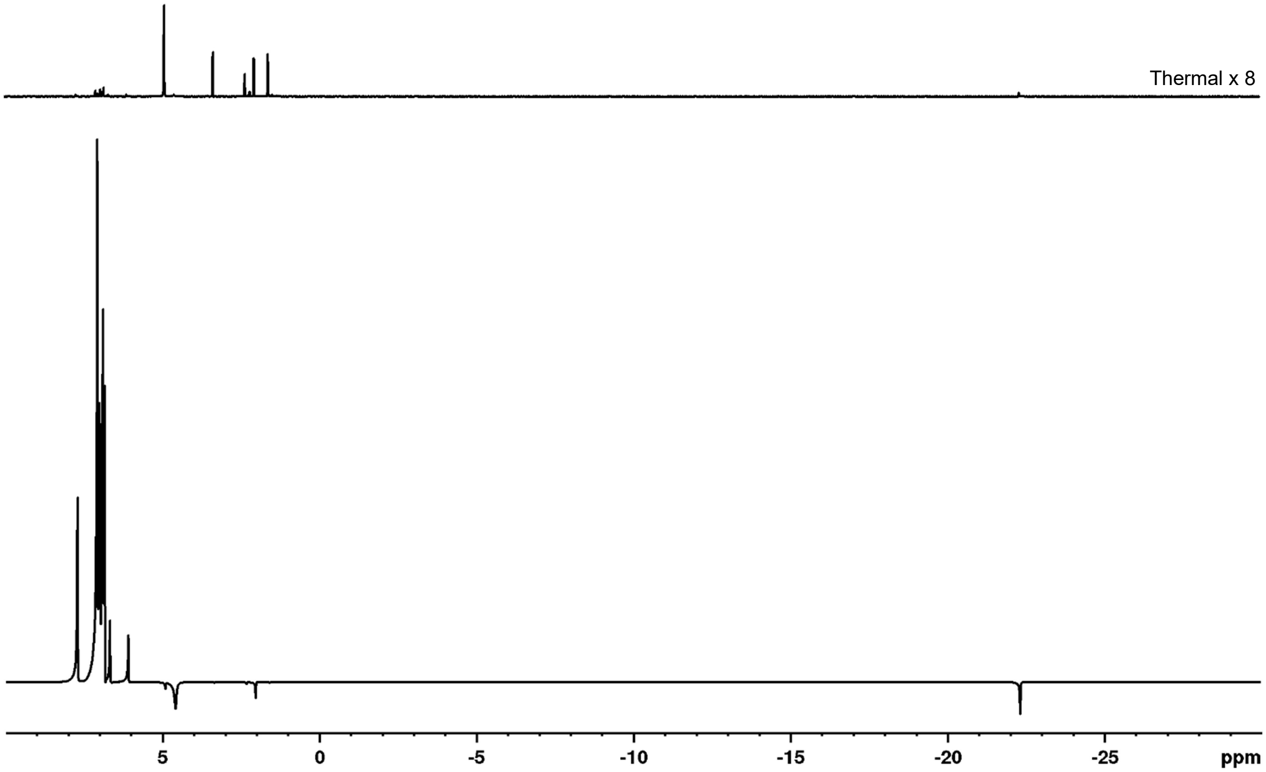

Figure S22: Hyperpolarised 1 scan ^{1}H NMR spectrum recorded at 298 K resulting from shaking a sample of [IrCl(COD)(IMes)], sodium pyruvate-1,2-[$^{13}C_2$] and imidazole in methanol-d_4 with 3 bar p-H_2 for 10 seconds at 65 G with corresponding thermal reference trace directly above.

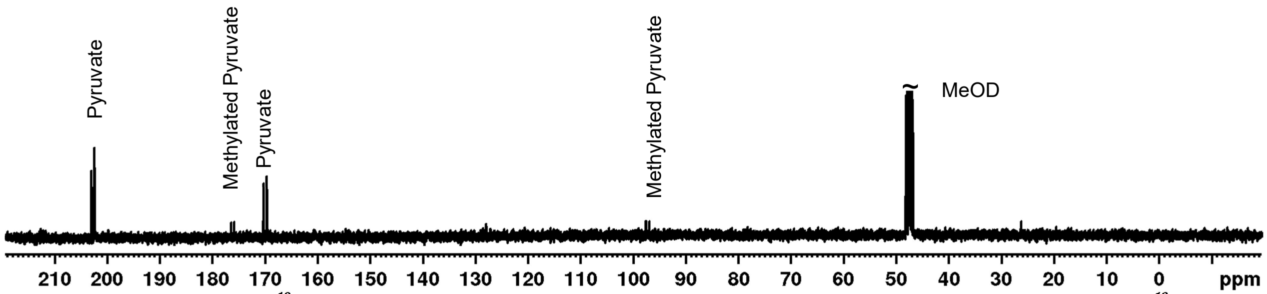

Figure S23: Thermal 128 scan ^{13}C NMR spectrum recorded at 298 K of a sample of [IrCl(COD)(IMes)], sodium pyruvate-1,2-[$^{13}C_2$] and imidazole in methanol-*d*$_4$ after leaving the sample for 120 mins in a water bath at 45 °C following the addition of 3 bar H_2.

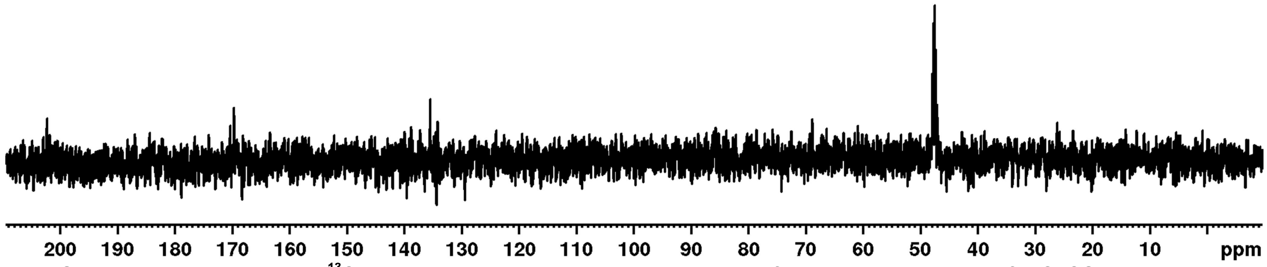

Figure S24: Hyperpolarised 1 scan ^{13}C NMR spectrum recorded at 298 K resulting from shaking a sample of [IrCl(COD)(IMes)], sodium pyruvate-1,2-[$^{13}C_2$] and imidazole in methanol-*d*$_4$ with 3 bar *p*-H_2 for 10 seconds in a mu metal shield.

S1.7: X-ray crystallography of $[Ir_2(H)_4(\kappa^2\text{-}SCH_2PhCl)_2(IMes)_2]$

Crystals were grown by leaving a sample containing 2 mg [IrCl(COD)(IMes)] (where IMes = 1,3-bis(2,4,6-trimethyl-phenyl)imidazol-2-ylidene and COD = *cis*,*cis*-1,5-cyclooctadiene) with 6 equivalents of sodium pyruvate-1,2-$[^{13}C_2]$ and 4 equivalents of 4-chlorobenzenemethanethiol in 0.6 mL of methanol-d_4 with 3 bar H_2 at 278 K for a period of several months. A suitable crystal was selected and mounted on an Oxford Diffraction SuperNova X-ray diffractometer. The crystal was kept at 110 K during data collection. Diffractometer control, data collection, initial unit cell determination, frame integration and unit-cell refinement was carried out with "CrysAlisPro".[2] Face-indexed absorption corrections were applied using spherical harmonics, implemented in SCALE3 ABSPACK scaling algorithm. Using Olex2,[3] the structure was solved with the ShelXT[4] structure solution program using Intrinsic Phasing and refined with the ShelXL[5] refinement package using Least Squares minimisation. X-ray crystal structures were deposited with the CCDC (deposition number 1957542-1957543)

The crystal of $[Ir_2(H)_4(\kappa^2\text{-}SCH_2PhCl)_2(IMes)_2]$ showed evidence of minor twinning with two residual density peaks close to the iridium atoms. This could not be resolved using either merohedral or non-merohedral twinning methods. One of the 4-chlorobenzyl groups was disordered and modelled in two positions with refined occupancies of 0.803:0.197(10). Pairs of disordered carbons were constrained to have the same ADP (e.g. C43, & C43a, C44 & C44a etc.). The S-CH_2 bond-lengths were restrained to be equal as were the CH_2-C(ipso) bond-lengths and the C-Cl bond-lengths. The phenyl ring of the minor form was constrained to be a regular hexagon with a C-C bond length of 1.39 angstroms. For the minor form the CH_2-C(ortho) distances were restrained to be equal as were the C(meta)-Cl distances. The hydrides were initially located by difference map, the Ir-H bond-length was then adjusted to be 1.65 angstroms and then the location fixed to ride on the iridium.

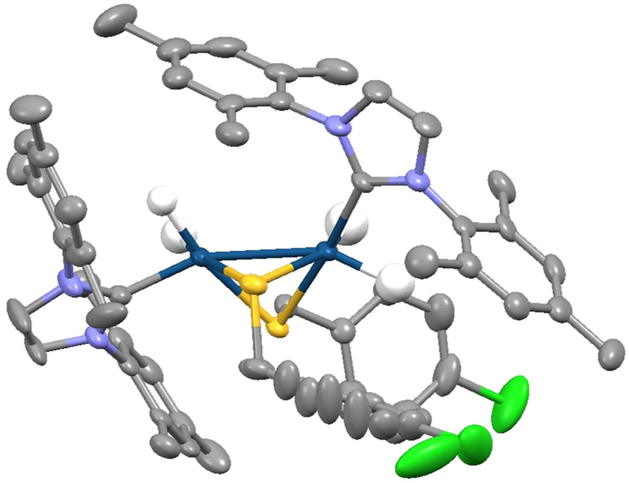

Figure S25: Structure of $[Ir_2(H)_4(\kappa^2\text{-}SCH_2PhCl)_2(IMes)_2]$ determined by X-ray diffraction studies. Note that all non hydride hydrogen atoms and solvent of crystallisation have been omitted for clarity

Table S1: Crystal data and structure refinement for $[Ir_2(H)_4(\kappa^2\text{-}SCH_2PhCl)_2(IMes)_2]$

Empirical formula	$C_{57}H_{68}Cl_2Ir_2N_4OS_2$
Formula weight	1344.57
Temperature/K	110.00(10)
Crystal system	monoclinic
Space group	$P2_1/c$
a/Å	12.9317(2)
b/Å	16.6000(3)
c/Å	25.5365(4)
α/°	90
β/°	90.2757(13)
γ/°	90
Volume/Å^3	5481.76(15)
Z	4

ρ_{calc}g/cm^3	1.629
μ/mm^{-1}	11.192
F(000)	2664.0
Crystal size/mm^3	0.149 × 0.09 × 0.078
Radiation	CuKα (λ = 1.54184)
2Θ range for data collection/°	6.924 to 134.152
Index ranges	-15 ≤ h ≤ 14, -19 ≤ k ≤ 18, -29 ≤ l ≤ 30
Reflections collected	20688
Independent reflections	9782 [R_{int} = 0.0256, R_{sigma} = 0.0331]
Data/restraints/parameters	9782/4/650
Goodness-of-fit on F^2	1.036
Final R indexes [I>=2σ (I)]	R_1 = 0.0299, wR_2 = 0.0678
Final R indexes [all data]	R_1 = 0.0357, wR_2 = 0.0712
Largest diff. peak/hole / e Å^{-3}	1.92/-1.36

S1.8: X-ray crystallography of [Ir(H)$_3$(PPh$_3$)$_3$]

Crystals were grown by leaving a sample containing 2 mg [IrCl(COD)(IMes)] (where IMes = 1,3-bis(2,4,6-trimethyl-phenyl)imidazole-2-ylidene and COD = *cis*,*cis*-1,5-cyclooctadiene) with 6 equivalents of sodium pyruvate-1,2-[$^{13}C_2$] and 4 equivalents of triphenylphosphine in 0.6 mL of methanol-*d*$_4$ with 3 bar H_2 at 278 K for a period of several months. A suitable crystal was selected and X-ray diffraction data was collected and solved as described in Section S1.7. The asymmetric unit contained a partial methanol whose occupancy refined to 0.283(5).

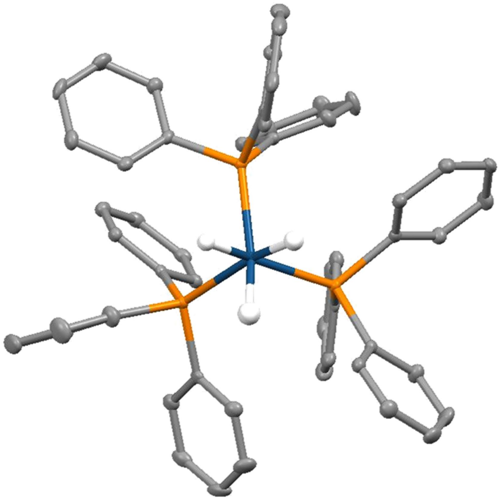

Figure S26: Structure of [Ir(H)$_3$(PPh$_3$)$_3$] determined by X-ray diffraction studies. Note that all non hydride hydrogen atoms and solvent of crystallisation have been omitted for clarity

Table S2: Crystal data and structure refinement for [Ir(H)$_3$(PPh$_3$)$_3$]

Empirical formula	$C_{54.28}H_{49.13}IrO_{0.28}P_3$
Formula weight	991.09
Temperature/K	110.00(10)
Crystal system	monoclinic
Space group	P2$_1$/c
a/Å	17.31801(12)
b/Å	12.99024(10)
c/Å	19.71788(16)
α/°	90
β/°	94.4442(7)

γ/°	90
Volume/Å^3	4422.50(6)
Z	4
ρ_{calc}g/cm^3	1.489
μ/mm^{-1}	7.149
F(000)	1996.4
Crystal size/mm^3	0.197 × 0.099 × 0.037
Radiation	CuKα (λ = 1.54184)
2Θ range for data collection/°	8.158 to 134.152
Index ranges	-15 ≤ h ≤ 20, -15 ≤ k ≤ 15, -23 ≤ l ≤ 21
Reflections collected	17542
Independent reflections	7896 [R_{int} = 0.0226, R_{sigma} = 0.0296]
Data/restraints/parameters	7896/0/557
Goodness-of-fit on F^2	1.047
Final R indexes [I>=2σ (I)]	R_1 = 0.0199, wR_2 = 0.0458
Final R indexes [all data]	R_1 = 0.0237, wR_2 = 0.0480
Largest diff. peak/hole / e Å^{-3}	0.71/-0.53

S2. Monitoring $^{13}C_2$ Pyruvate signal enhancement over time

S2.1: Effect of changing the sulfoxide identity

Dimethylsulfoxide (DMSO) (**I**), phenylmethylsulfoxide (**II**), chlorophenylmethylsulfoxide (**III**), vinylsulfoxide (**IV**), diphenylsulfoxide (**V**), dibenzylsulfoxide (**VI**), dibutylsulfoxide (**VII**), tetramethylene sulfoxide (**VIII**), methionine sulfoxide (**IX**) and Fmoc-L-methionine sulfoxide (**X**) were used in this work. Their structures are given in Figure 2 of the main manuscript.

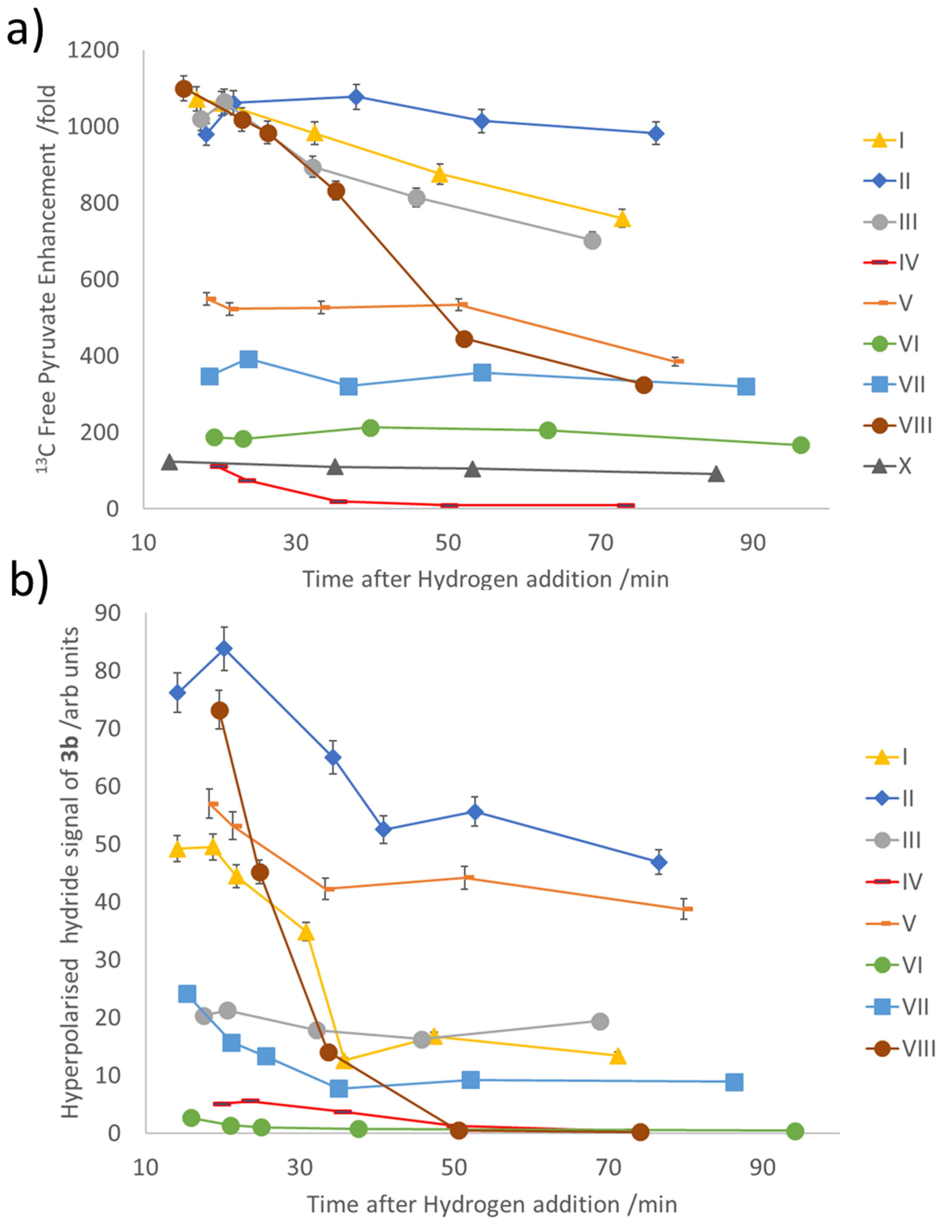

Figure S27: Results after shaking a sample of [IrCl(COD)(IMes)], 6 equivalents of sodium pyruvate-1,2-[$^{13}C_2$], and 4 equivalents of the specified sulfoxide I-X in 0.6 mL methanol-*d*$_4$ with 3 bar p-H_2 a) averaged $^{13}C_2$ pyruvate enhancement and b) hyperpolarised ^1H 3b hydride signal intensities monitored over the first 90 minutes of reaction following initial H_2 addition.

The concentration of methylphenylsulfoxide **II** was varied to determine its effect on pyruvate enhancement. The relative ^{13}C NMR signal gains for bound and free pyruvate, in addition to the hydride ligand signal enhancements for its **3b** derivative across a range of concentrations, are presented in Figure 3

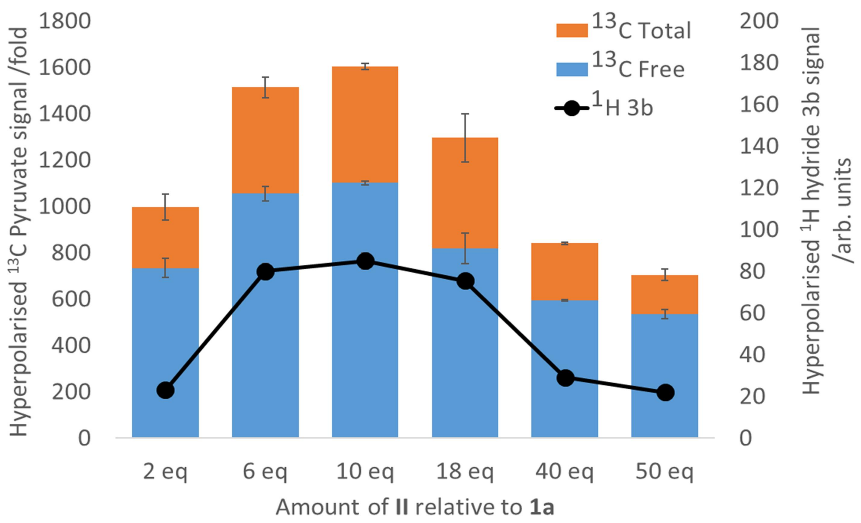

Figure S28: Averaged hyperpolarised ^{13}C pyruvate (left axis, bars) and ^{1}H hydride signals of 3b derivative (right axis, line) detected as a function of sulfoxide concentration after a methanol-*d_4* solution of 1a, 6 equivalents of sodium pyruvate-1,2-[$^{13}C_2$] and the indicated equivalents of II are shaken with 3 bar *p*-H_2 for 10 seconds in a mu metal shield.

S2.2: Effect of changing the chloride concentration

Solutions of 2 mg **1a,** 10 equivalents of sulfoxide **I** and 5 equivalents of sodium pyruvate-1,2-[$^{13}C_2$] in 0.6 mL methanol-d_4 containing 0, 1, 3 or 5 equivalents of NaCl in 5 µL of D_2O were prepared. These four solutions were activated with 3 bar H_2 and their $^{13}C_2$ pyruvate enhancement monitored as a function of reaction time. Signal enhancements for this data were calculated by reference to a thermal 128 scan ^{13}C NMR spectrum of the same sample and were consistent with those calculated by reference to a more concentrated sodium pyruvate-1,2-[$^{13}C_2$] thermal sample as outlined in Shchepin *et* al.[6]

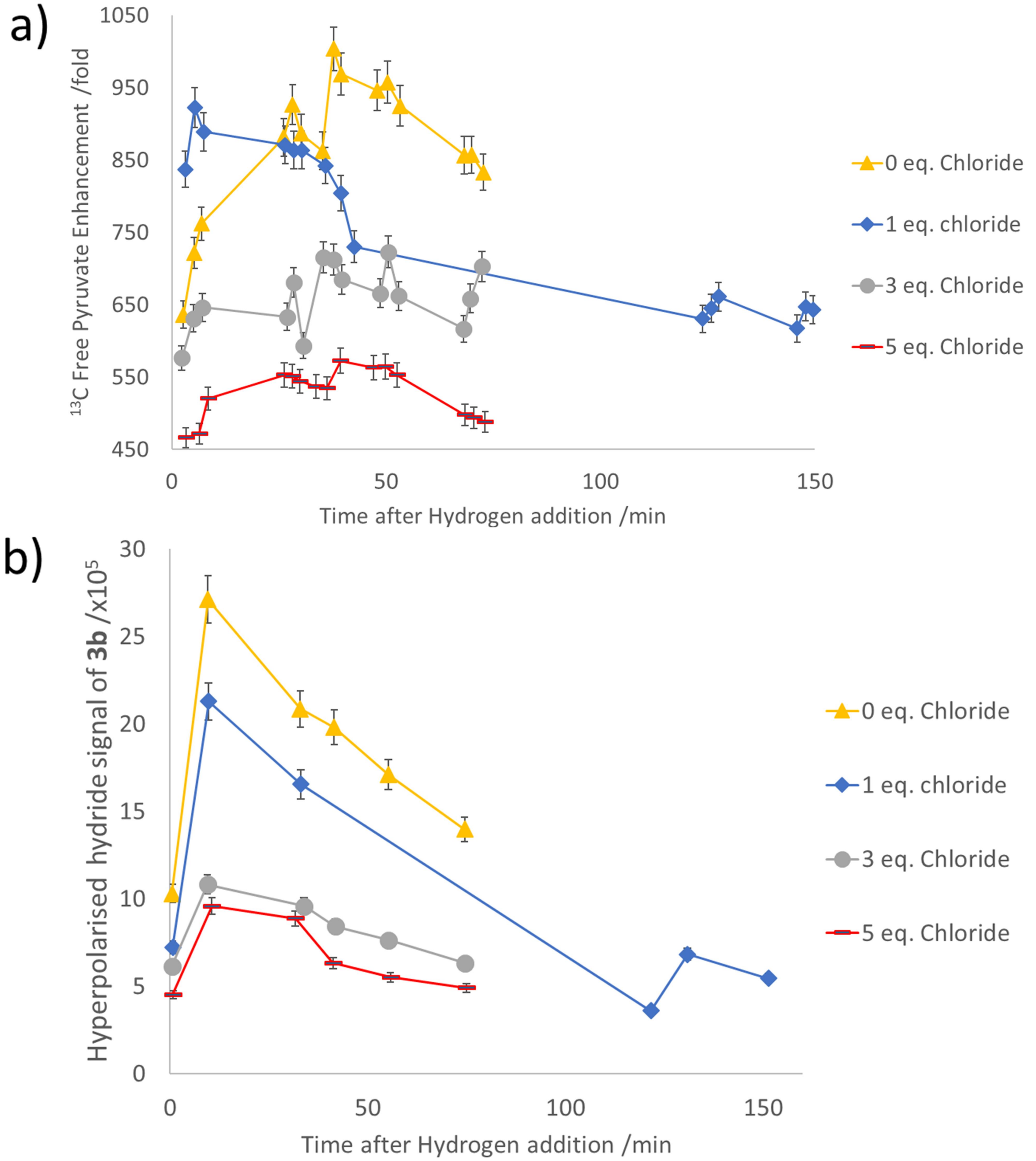

Figure S29: Upon shaking a sample of [IrCl(COD)(IMes)], 5 equivalents of sodium pyruvate-1,2-[$^{13}C_2$], and 10 equivalents of DMSO with varying amounts of NaCl in 0.6 mL methanol-d_4 with 3 bar p-H_2 the size of the a) average $^{13}C_2$ pyruvate enhancement and b) hyperpolarised ^1H 3b hydride signal intensities were monitored following initial H_2 addition.

S2.3: Effect of changing the carbene ligand

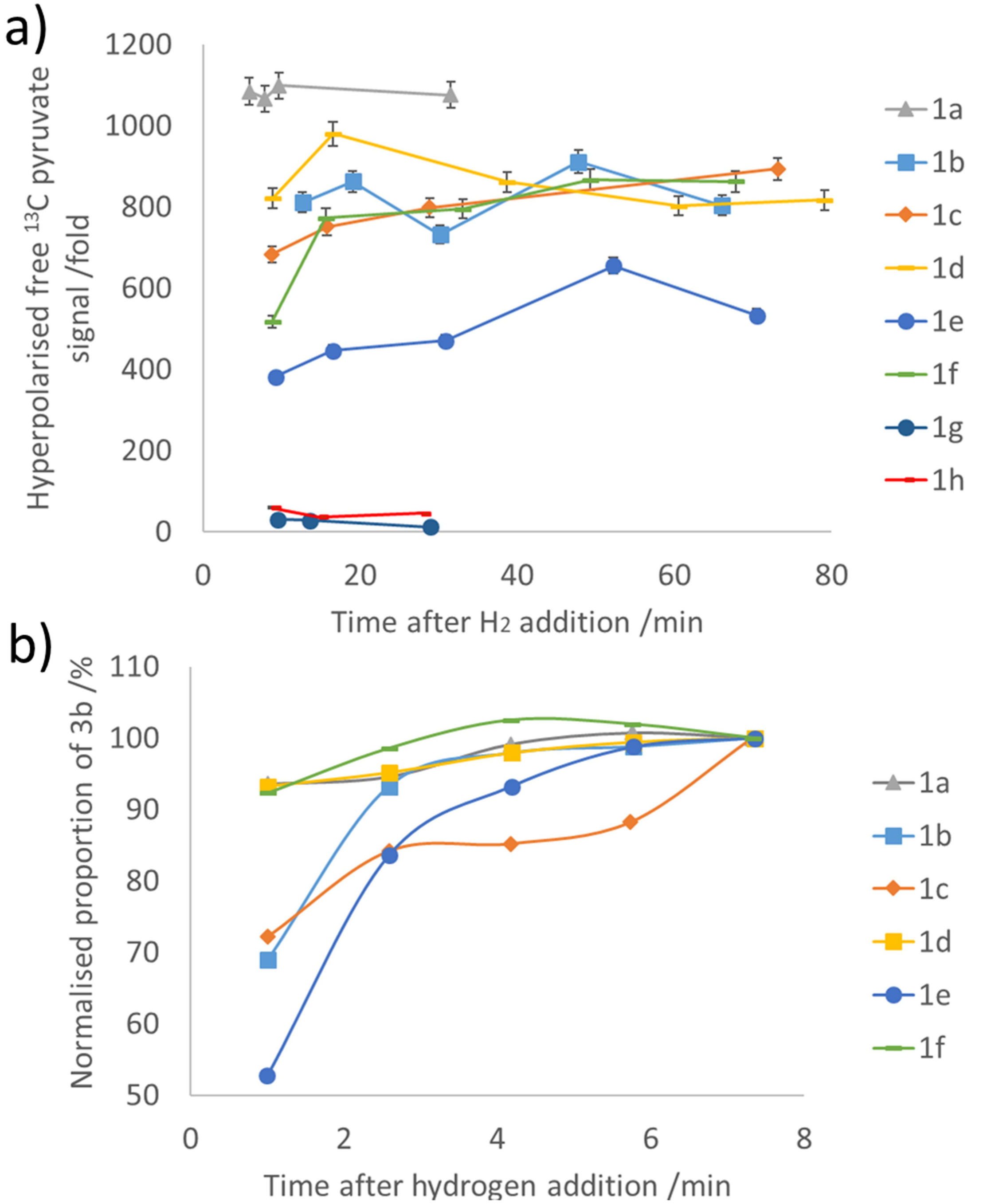

Figure S30 a) Average hyperpolarised $^{13}C_2$ pyruvate responses and b) proportion of 3b relative to all other hydride containing species when a sample of the iridium precatalyst 1a-h, 6 equivalents of sodium pyruvate-1,2-[$^{13}C_2$], and 4 equivalents of methylphenylsulfoxide is shaken in 0.6 mL methanol-*d*$_4$ with 3 bar *p*-H_2 for 10 seconds in a mu metal shield and then monitored periodically after this point.

S3. Hyperpolarised ^{13}C and ^{1}H NMR spectra

S3.1: Typical hyperpolarised ^{13}C and ^{1}H NMR spectra

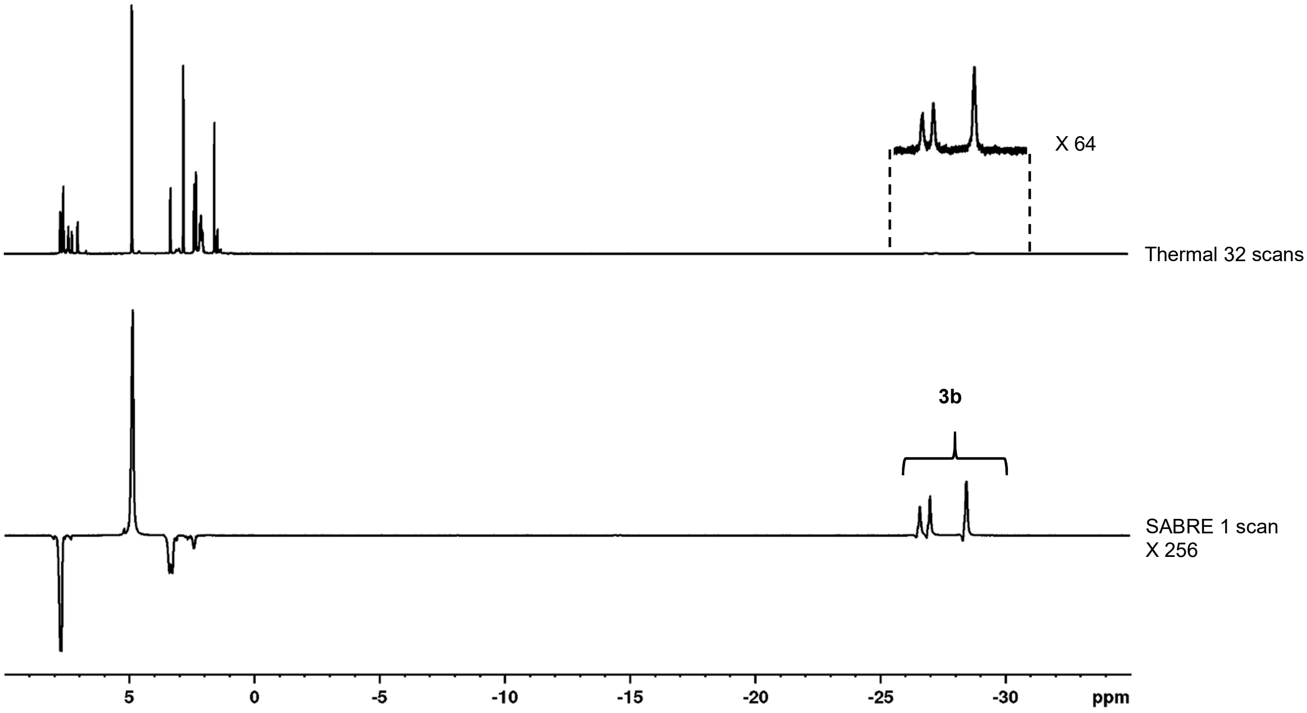

Figure S31: Hyperpolarised 1 scan ^{1}H NMR spectrum recorded at 298 K (below) resulting from shaking a sample of [IrCl(COD)(IMes)], sodium pyruvate-1,2-[$^{13}C_2$] and phenylmethylsulfoxide in methanol-d_4 with 3 bar p-H_2 for 10 seconds at 65 G with the corresponding thermal measurement (32 scans) displayed above.

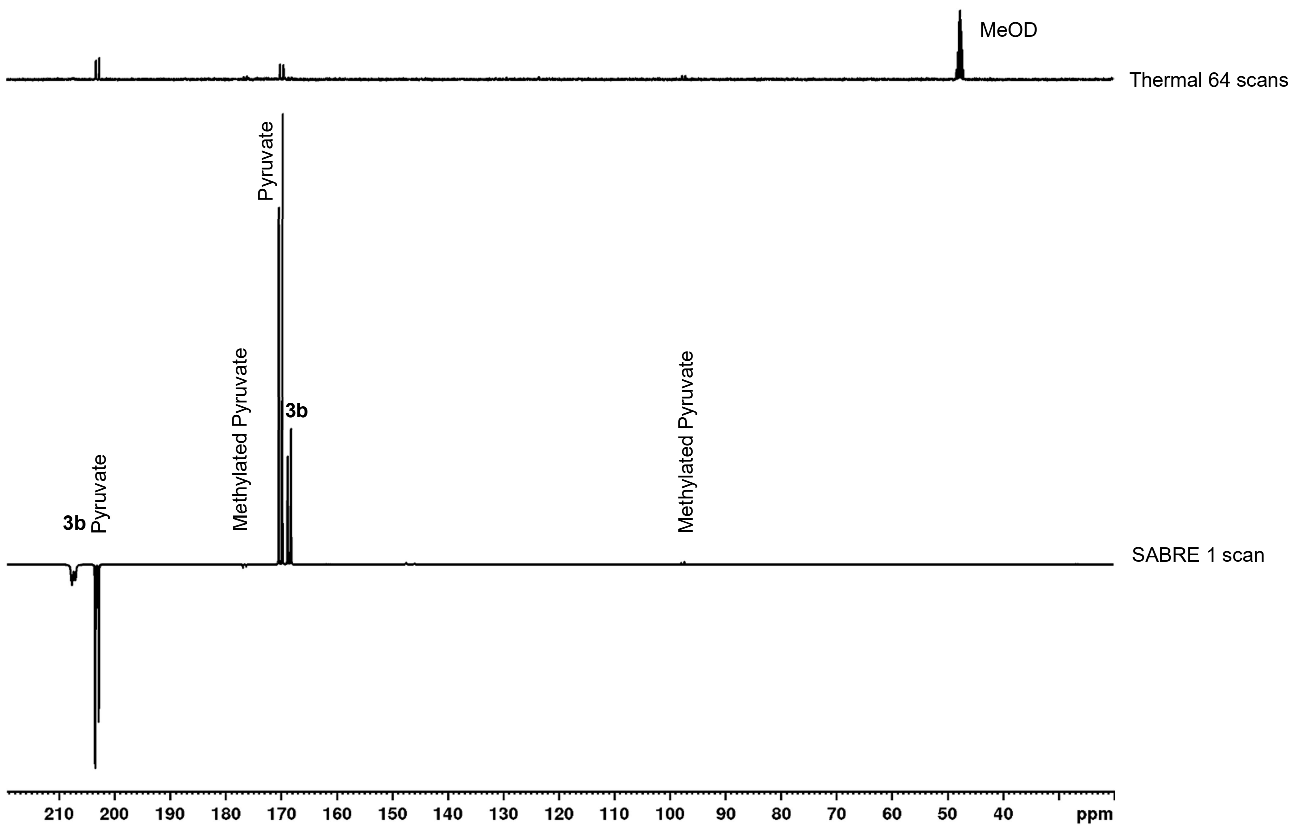

Figure S32: Hyperpolarised 1 scan ^{13}C NMR spectrum recorded at 298 K resulting from shaking a sample of of [IrCl(COD)(IMes)], sodium pyruvate-1,2-[$^{13}C_2$] and phenylmethylsulfoxide in methanol-d_4 with 3 bar p-H_2 for 10 seconds in a mu metal shield 65 G with the corresponding thermal measurement (64 scans) displayed above.

S3.2: Hyperpolarised ^{13}C and ^{1}H spectra using sulfoxide IX

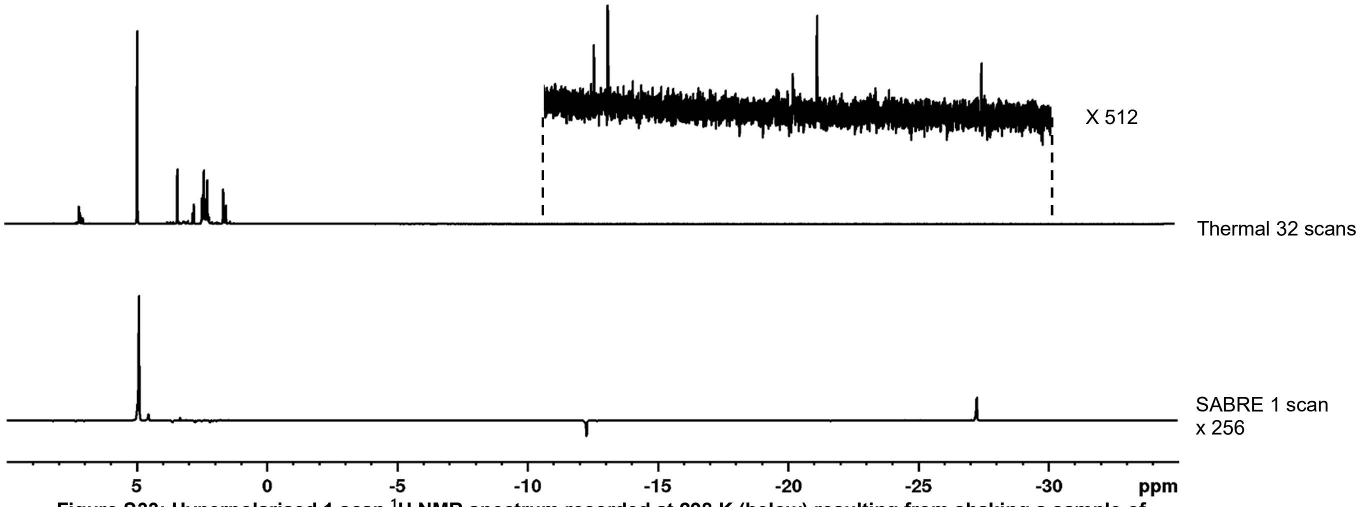

Figure S33: Hyperpolarised 1 scan ^{1}H NMR spectrum recorded at 298 K (below) resulting from shaking a sample of [IrCl(COD)(IMes)], sodium pyruvate-1,2-[$^{13}C_2$] and sulfoxide IX in methanol-d_4 with 3 bar p-H_2 for 10 seconds at 65 G with the corresponding thermal measurement (64 scans) displayed above.

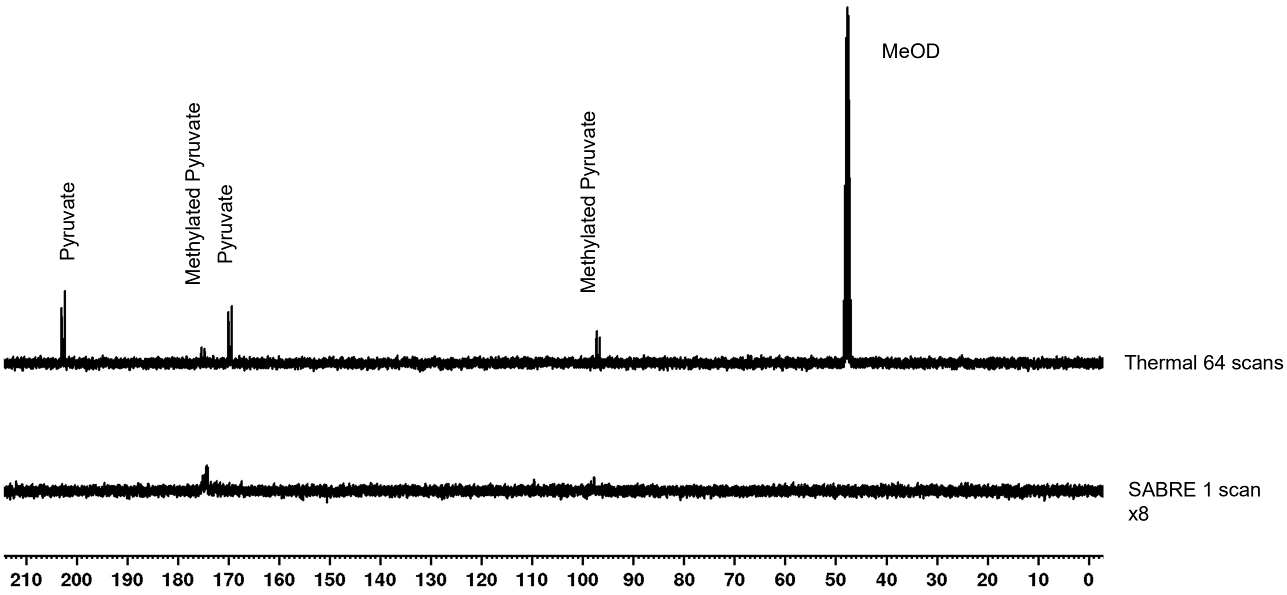

Figure S34: Hyperpolarised 1 scan ^{13}C NMR spectrum recorded at 298 K (below) resulting from shaking a sample of [IrCl(COD)(IMes)], sodium pyruvate-1,2-[$^{13}C_2$] and sulfoxide IX in methanol-*d*$_4$ with 3 bar *p*-H_2 for 10 seconds in a mu metal shield with the corresponding thermal measurement (64 scans) displayed above.

S3.3: Hyperpolarised ^{13}C and ^{1}H spectra using sulfoxide X

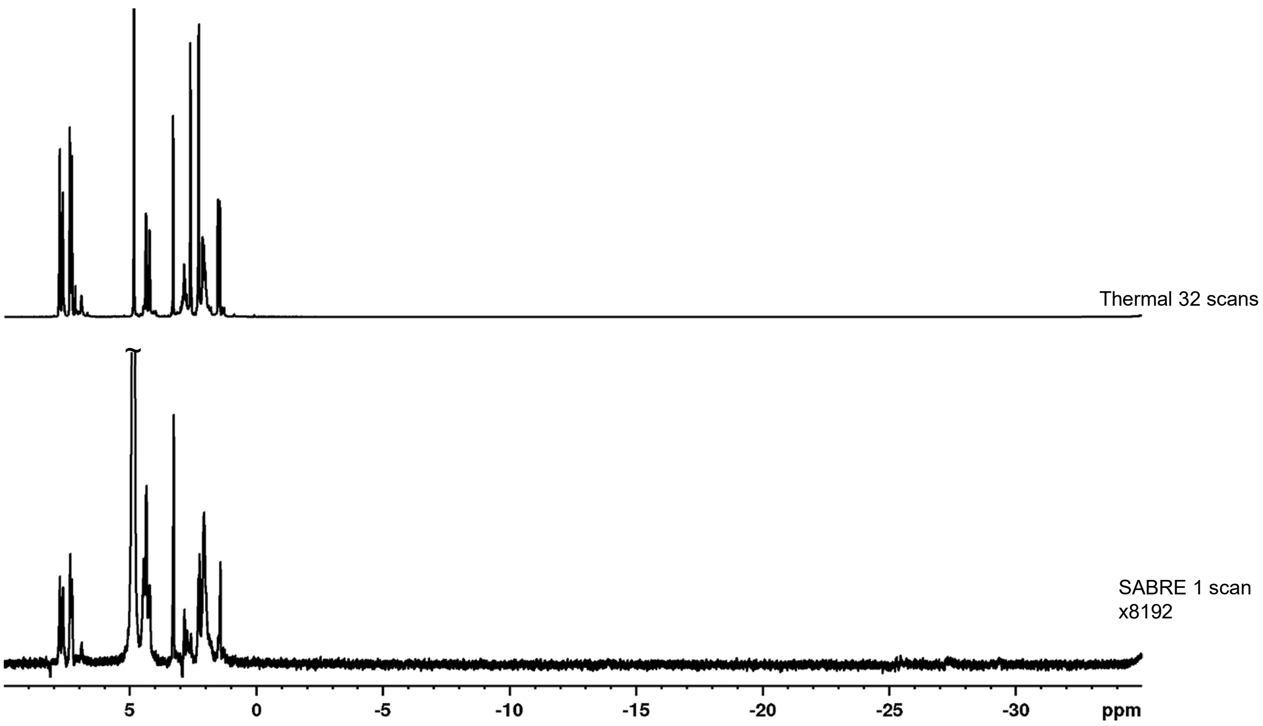

Figure S35: Hyperpolarised 1 scan ^{1}H NMR spectrum recorded at 298 K (below) resulting from shaking a sample of [IrCl(COD)(IMes)], sodium pyruvate-1,2-[$^{13}C_2$] and sulfoxide X in methanol-*d*$_4$ with 3 bar *p*-H_2 for 10 seconds at 65 G with the corresponding thermal measurement (64 scans) displayed above.

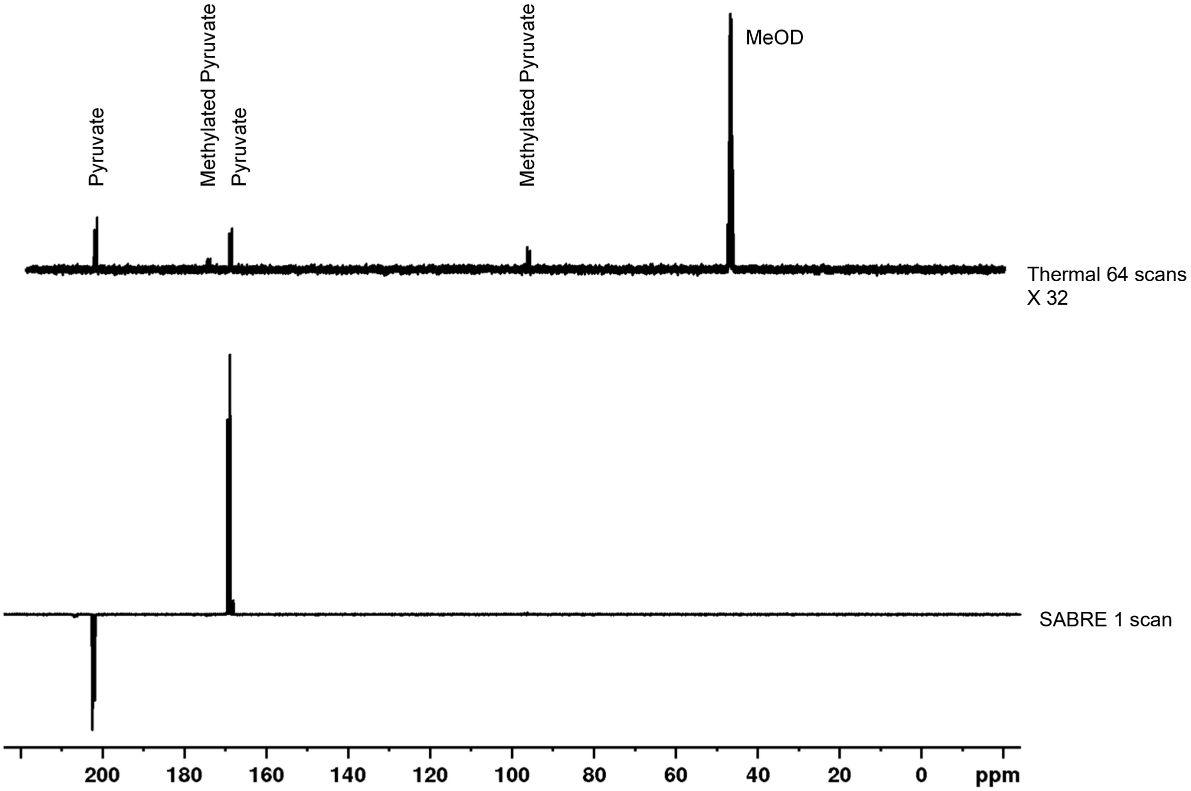

Figure S36: Hyperpolarised 1 scan ^{13}C NMR spectrum recorded at 298 K (below) resulting from shaking a sample of [IrCl(COD)(IMes)], sodium pyruvate-1,2-[$^{13}C_2$] and sulfoxide X in methanol-d_4 with 3 bar p-H_2 for 10 seconds in a mu metal shield with the corresponding thermal measurement (64 scans) displayed above.

S4. Optimisation of $^{13}C_2$ Pyruvate signal enhancement

S4.1: Effect of shaking time and *p*-H_2 pressure

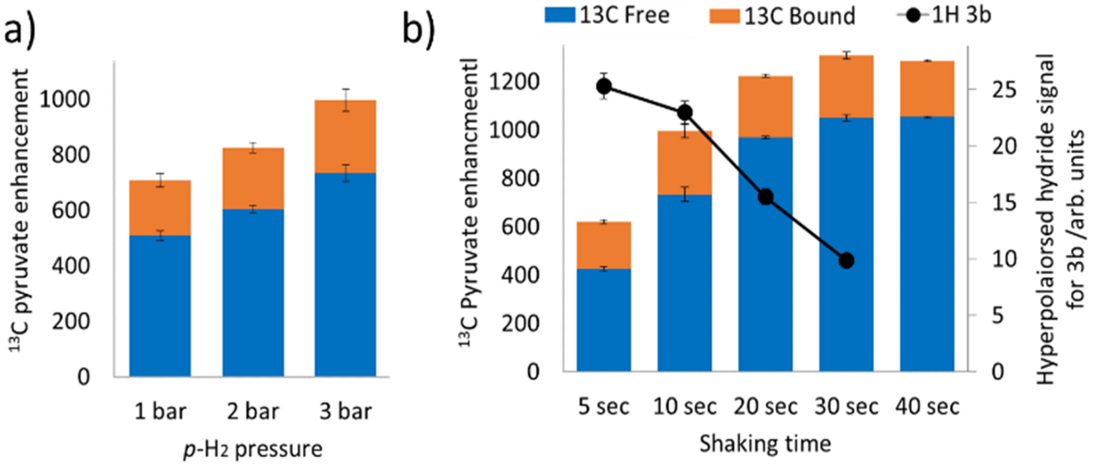

Figure S37: a) Average hyperpolarised ^{13}C pyruvate signal enhancement as a function of hydrogen pressure (1a with 2 eq phenylmethylsulfoxide, 3 bar p-H_2 and 10 second shaking). Hyperpolarised b) ^{13}C pyruvate and ^{1}H hydride responses seen for 3b as a function of shaking time recorded on the same sample (3 bar).

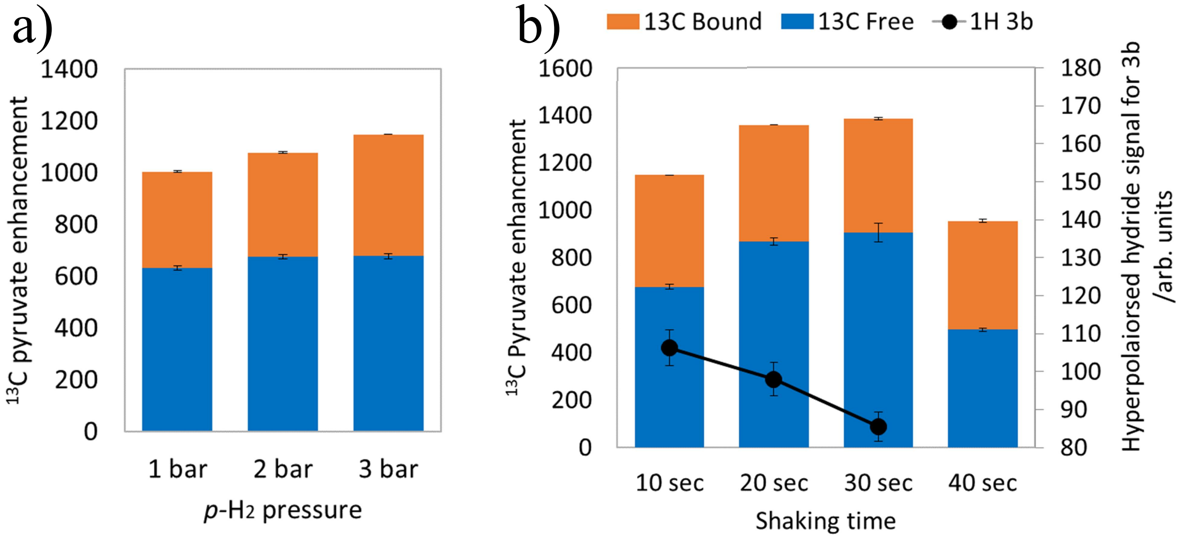

Figure S38: a) Average hyperpolarised ^{13}C pyruvate signal enhancement as a function of hydrogen pressure (1a-d_{24} with 10 eq phenylmethylsulfoxide, 3 bar p-H_2 and 10 second shaking). Hyperpolarised b) ^{13}C pyruvate and ^{1}H hydride responses seen for 3b as a function of shaking time recorded on the same sample (3 bar).

S4.2: Effect of pyruvate concentration

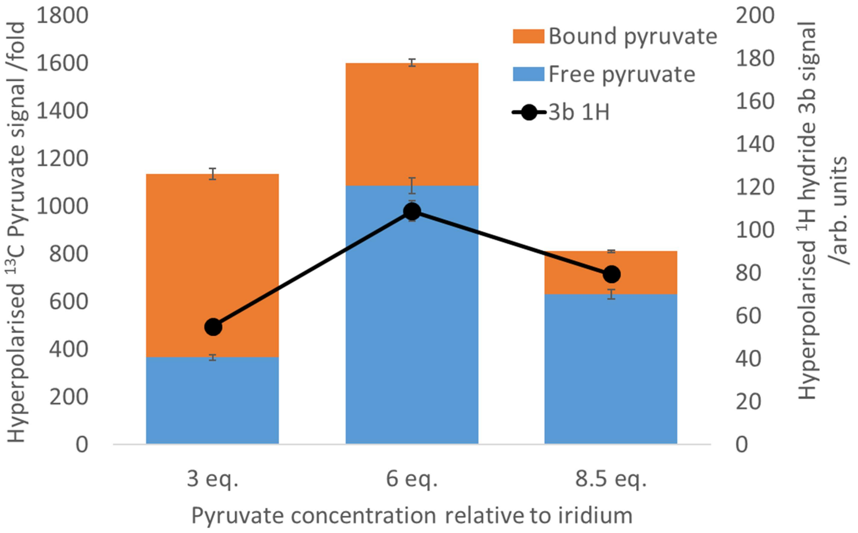

Figure S39: Average hyperpolarised ^{13}C pyruvate signal enhancement and hypeprolarised hydride signal intensity of 3b as a function of pyruvate concentration relative to iridium for a sample containing 1a with 10 eq phenylmethylsulfoxide and 3 bar p-H_2 after 10 seconds of shaking.

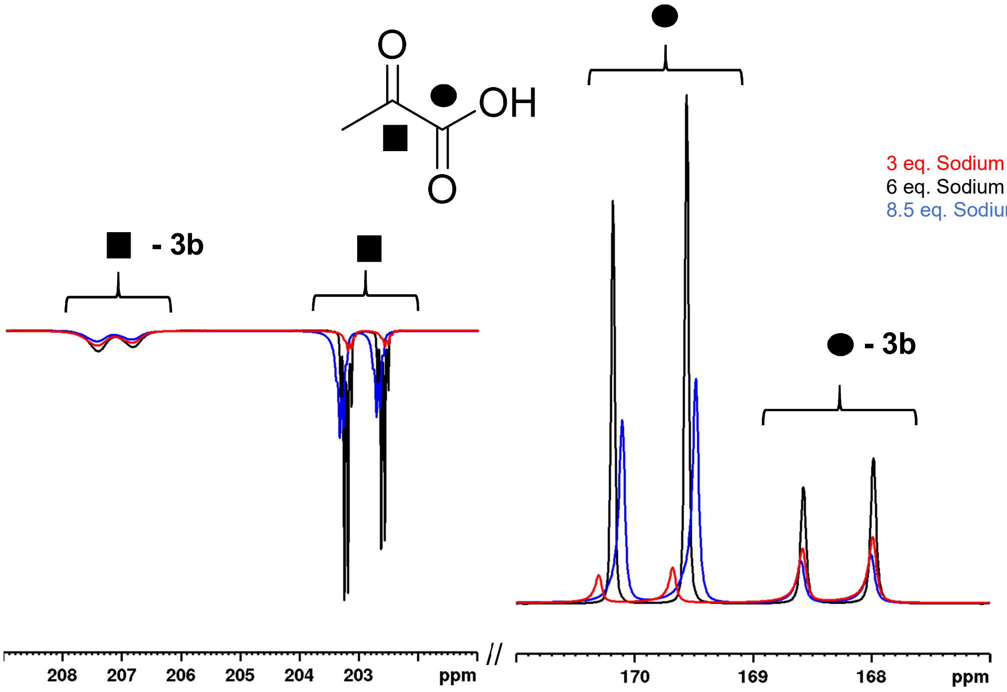

Figure S40: Partial ^{13}C hyperpolarised NMR spectra resulting from shaking a sample of [IrCl(COD)(IMes)] with the indicated equivalents of sodium pyruvate-1,2-[$^{13}C_2$] and 10 equivalents of methylphenylsulfoxide in methanol-*d*$_4$ with 3 bar p-H_2 for 10 seconds in a mu metal shield at similar time points after the initial H_2 addition step.

S5. References

1. L. D. Vazquez-Serrano, B. T. Owens and J. M. Buriak, *Inorg. Chim. Acta*, 2006, **359**, 2786-2797.
2. CrysAlisPro, *Oxford Diffraction Ltd.*, Version 1.171.34.41.
3. O. V. Dolomanov, L. J. Bourhis, R. J. Gildea, J. A. Howard and H. Puschmann, *J. Appl. Cryst.*, 2009, **42**, 339-341.
4. G. Sheldrick, *Acta Crys. Sec. A.*, 2015, **71**, 3-8.
5. G. Sheldrick, *Acta Crys. Sec. C.*, 2015, **71**, 3-8.
6. R. V. Shchepin, L. Jaigirdar, T. Theis, W. S. Warren, B. M. Goodson and E. Y. Chekmenev, *J. Phys. Chem. C*, 2017, **121**, 28425-28434.
